# TL1A is an epithelial alarmin that cooperates with IL-33 for initiation of allergic airway inflammation

**DOI:** 10.1084/jem.20231236

**Published:** 2024-04-10

**Authors:** Pauline Schmitt, Anais Duval, Mylène Camus, Emma Lefrançais, Stéphane Roga, Cécile Dedieu, Nathalie Ortega, Elisabeth Bellard, Emilie Mirey, Emmanuelle Mouton-Barbosa, Odile Burlet-Schiltz, Anne Gonzalez-de-Peredo, Corinne Cayrol, Jean-Philippe Girard

**Affiliations:** 1 Institut de Pharmacologie et de Biologie Structurale (IPBS), Université de Toulouse, CNRS, Université Toulouse III—Paul Sabatier (UPS), Toulouse, France

## Abstract

Epithelium-derived cytokines or alarmins, such as interleukin-33 (IL-33) and thymic stromal lymphopoietin (TSLP), are major players in type 2 immunity and asthma. Here, we demonstrate that TNF-like ligand 1A (TL1A) is an epithelial alarmin, constitutively expressed in alveolar epithelium at steady state in both mice and humans, which cooperates with IL-33 for early induction of IL-9^high^ ILC2s during the initiation of allergic airway inflammation. Upon synergistic activation by IL-33 and TL1A, lung ILC2s acquire a transient IL-9^high^GATA3^low^ “ILC9” phenotype and produce prodigious amounts of IL-9. A combination of large-scale proteomic analyses, lung intravital microscopy, and adoptive transfer of ILC9 cells revealed that high IL-9 expression distinguishes a multicytokine-producing state-of-activated ILC2s with an increased capacity to initiate IL-5-dependent allergic airway inflammation. Similar to IL-33 and TSLP, TL1A is expressed in airway basal cells in healthy and asthmatic human lungs. Together, these results indicate that TL1A is an epithelium-derived cytokine and an important cofactor of IL-33 in the airways.

## Teaser

Schmitt, Duval, et al. show that TL1A is an epithelial cytokine that cooperates with IL-33 in the initiation of allergic airway inflammation. Similar to IL-33, TL1A functions as an alarmin important for early induction of IL-9^high^ ILC2s after allergen exposure.

## Graphical Abstract

**Figure:**
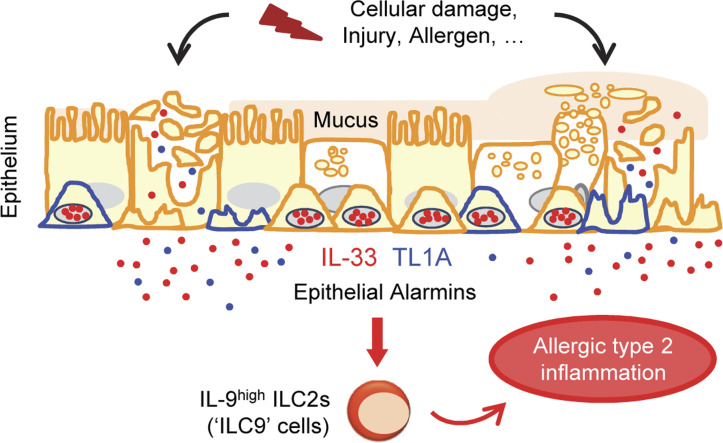

## Introduction

Allergic inflammation plays crucial roles in allergic diseases such as asthma and allergic rhinitis (Hammad and Lambrecht, 2021; Locksley, 2010). Severe asthmatic disease may cause a reduced quality of life and lead to premature death. It is therefore important to understand the mechanisms that contribute to allergic airway inflammation and asthma. Advances in the last 15 years have considerably changed our view of barrier epithelial cells, and the airway epithelium is now recognized as a major player in type 2 immunity and asthma (Hammad and Lambrecht, 2021; Howell et al., 2023; Lambrecht et al., 2019; Lloyd and Snelgrove, 2018; Locksley, 2010; Molofsky and Locksley, 2023). In response to various stimuli that induce type 2 responses and damage the airways, epithelial cells release prototypical cytokines such as interleukin-33 (IL-33) and thymic stromal lymphopoietin (TSLP) (Cayrol and Girard, 2018; Hammad and Lambrecht, 2021; Lambrecht et al., 2019; Lloyd and Snelgrove, 2018; Locksley, 2010; Molofsky and Locksley, 2023). Genome-wide association studies and successful phase 2/phase 3 clinical trials support the critical role of IL-33 and TSLP in human asthma (Cayrol and Girard, 2014, 2022; Corren et al., 2017; Howell et al., 2023; Menzies-Gow et al., 2021; Wechsler et al., 2021). IL-33, a member of the IL-1 cytokine family, is a chromatin-associated nuclear cytokine abundantly expressed in epithelial cells of barrier tissues, endothelial cells of blood vessels, and fibroblastic stromal cells in various tissues (Cayrol and Girard, 2018, 2022; Moussion et al., 2008; Pichery et al., 2012). It functions as an alarm signal or alarmin cytokine, expressed constitutively in producing cells and rapidly released upon cellular damage or tissue injury (Cayrol and Girard, 2009; Luthi et al., 2009), to alert tissue-resident immune cells expressing the ST2 (IL1RL1) receptor, such as mast cells and group 2 innate lymphoid cells (ILC2s) (Cayrol and Girard, 2018; Topczewska et al., 2023). Although full-length IL-33 (IL-33_FL_) is biologically active, inflammatory and allergen proteases generate shorter mature forms of the cytokine that have highly increased biological activity (Cayrol, 2021; Cayrol et al., 2018; Cayrol and Girard, 2022; Lefrançais et al., 2014; Lefrancais et al., 2012; Scott et al., 2018).

Environmental airborne allergens, including house dust mites, fungi, and pollens, are central to the development of allergic asthma (Locksley, 2010). The widely distributed fungus *Alternaria alternata* is one of the major aeroallergens associated with the development and persistence of allergic inflammation and asthma (Bush and Prochnau, 2004; Downs et al., 2001; Halonen et al., 1997; O’Hollaren et al., 1991; Pulimood et al., 2007). Dispersion of *A. alternata* spores during warm, dry, and windy weather conditions is associated with epidemic asthma, rapid onset life-threatening exacerbations, and increased mortality (Bush and Prochnau, 2004; Pulimood et al., 2007). The IL-33/ST2 pathway is rapidly activated after exposure to *A. alternata* and initiates allergic inflammation through stimulation of IL-5 and IL-13 production by lung ILC2s and induction of lung eosinophilia (Bartemes et al., 2012; Cayrol et al., 2018; Doherty et al., 2012; Kouzaki et al., 2011; McSorley et al., 2014; Scott et al., 2018; Snelgrove et al., 2014).

In addition to IL-33, many other mediators can induce IL-5 and IL-13 expression in ILC2s (Kabata et al., 2018; Rodriguez-Rodriguez et al., 2021), including other epithelial cytokines (TSLP, IL-25), lipid mediators, neuropeptides, and TNF-like ligand 1A (TL1A encoded by *TNFSF15*) (Meylan et al., 2014; Yu et al., 2014), a type II transmembrane protein from the TNF family (Meylan et al., 2011; Migone et al., 2002; Richard et al., 2015). Human and mouse ILC2s constitutively express the TL1A receptor DR3 (Meylan et al., 2014; Yu et al., 2014). DR3, a member of the TNF receptor superfamily, contains a death domain, like TNFR1, and signals through the adapter protein TRADD, which recruits TRAF2 and RIP, for the activation of NF-κB and MAP kinases pathways (Chinnaiyan et al., 1996; Meylan et al., 2011). Lung ILC2s also express high levels of the IL-9 receptor (IL-9R) and are major targets of IL-9 through autocrine or paracrine signaling (Mohapatra et al., 2016; Price et al., 2010; Turner et al., 2013; Wilhelm et al., 2011). Indeed, ILC2s can produce IL-9 in an IRF4-dependent manner (Mohapatra et al., 2016), and IL-9-mediated autocrine signaling through IL-9R has been shown to promote IL-5 and IL-13 expression and survival of ILC2s in vivo (Licona-Limon et al., 2013; Mohapatra et al., 2016; Turner et al., 2013; Wilhelm et al., 2011).

In an effort to better understand the mechanisms involved in the initiation of allergic inflammation, we searched for IL-33 cofactors that could act upstream in lung inflammatory cascades at the level of the epithelium. Here, we demonstrate that TL1A is an epithelial cytokine constitutively expressed in human and mouse lung epithelium that functions similarly to IL-33, as an alarmin rapidly released after a single exposure to *A. alternata*. We show that endogenous TL1A cooperates with IL-33 for early induction of IL-9^high^ ILC2s during the onset of allergic airway inflammation. Large-scale proteomic and kinetic analyses revealed that after synergistic activation by IL-33 and TL1A, lung ILC2s acquire a transient IL-9^high^GATA3^low^ “ILC9” phenotype, characterized by simultaneous production of large amounts of IL-9, IL-5, and IL-13. ILC9 cells have an increased capacity to persist in vivo in an activated state and to initiate IL-5-dependent allergic airway inflammation compared with “classical” IL-33-activated ILC2s. We propose that epithelial alarmins IL-33 and TL1A, and IL-9^high^ ILC2s function together in a potent alarm system that is activated after a single allergen exposure for the initiation of airway inflammation.

## Results

### TL1A is an epithelial cytokine expressed in alveolar epithelium and airway basal cells in healthy and asthmatic human lungs

**Figure 1.**
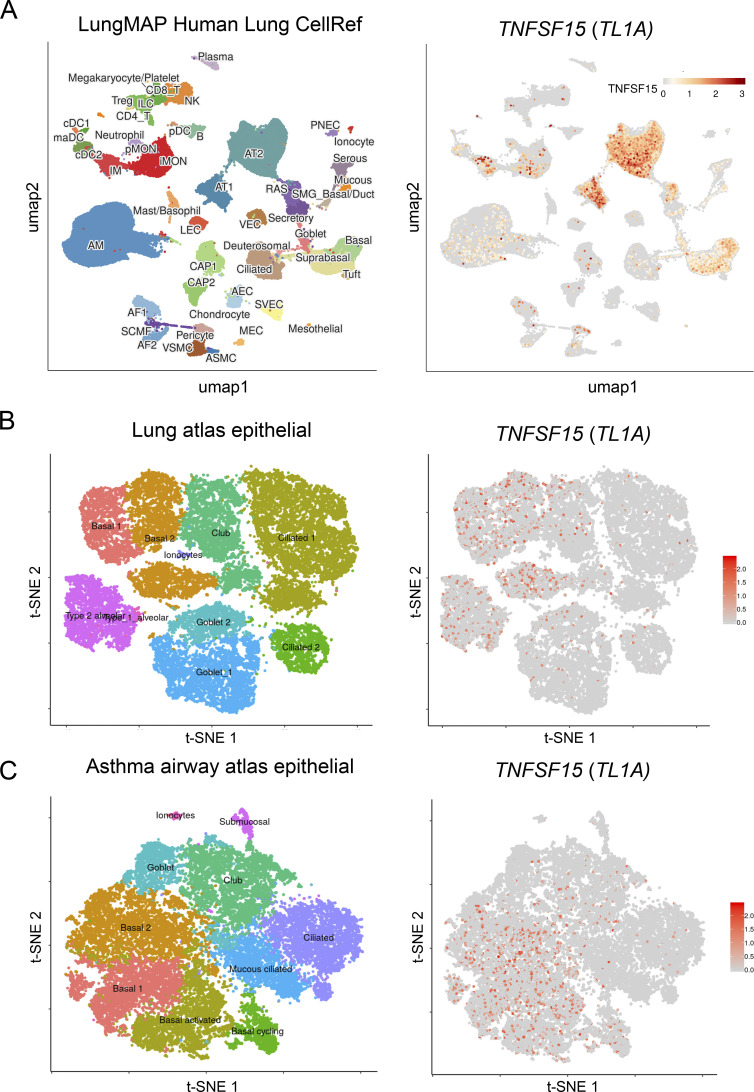
**TL1A is an epithelial cytokine expressed in alveolar epithelium and airway basal cells in human healthy and asthmatic lungs. (A)** Single-cell RNA-seq analysis of *TNFSF15* (*TL1A*) expression in the LungMAP single-cell human lung atlas. Uniform manifold projection (UMAP) plots show the clustering of 347,970 lung cells (10 single-cell datasets, 148 normal human lung samples from 104 donors: adult, child, and adolescent). Results are visualized using ShinyCell (Ouyang et al., 2021) and are based upon data generated by the LungMAP Consortium (Guo et al., 2023) and downloaded from http://www.lungmap.net (Gaddis et al., 2024). **(B and C)** Single-cell RNA-seq analysis of *TNFSF15* (*TL1A*) expression in epithelial cells from human healthy (B) and asthmatic (C) lungs. t-SNE plots show clustering of 26,154 epithelial cells in upper and lower airways and lung parenchyma in healthy lungs (B; 17 human samples: 6 alveoli and parenchyma, 9 bronchi, 2 nasal), and 25,146 epithelial cells from lower airways in healthy and asthmatic lungs (C; 12 human samples: 15,033 cells from 6 asthma bronchi; 10,113 cells from 6 control bronchi). t-SNE plots were extracted from data obtained by the human lung single-cell atlas (Vieira Braga et al., 2019) and downloaded from https://asthma.cellgeni.sanger.ac.uk.

**Figure S1.**
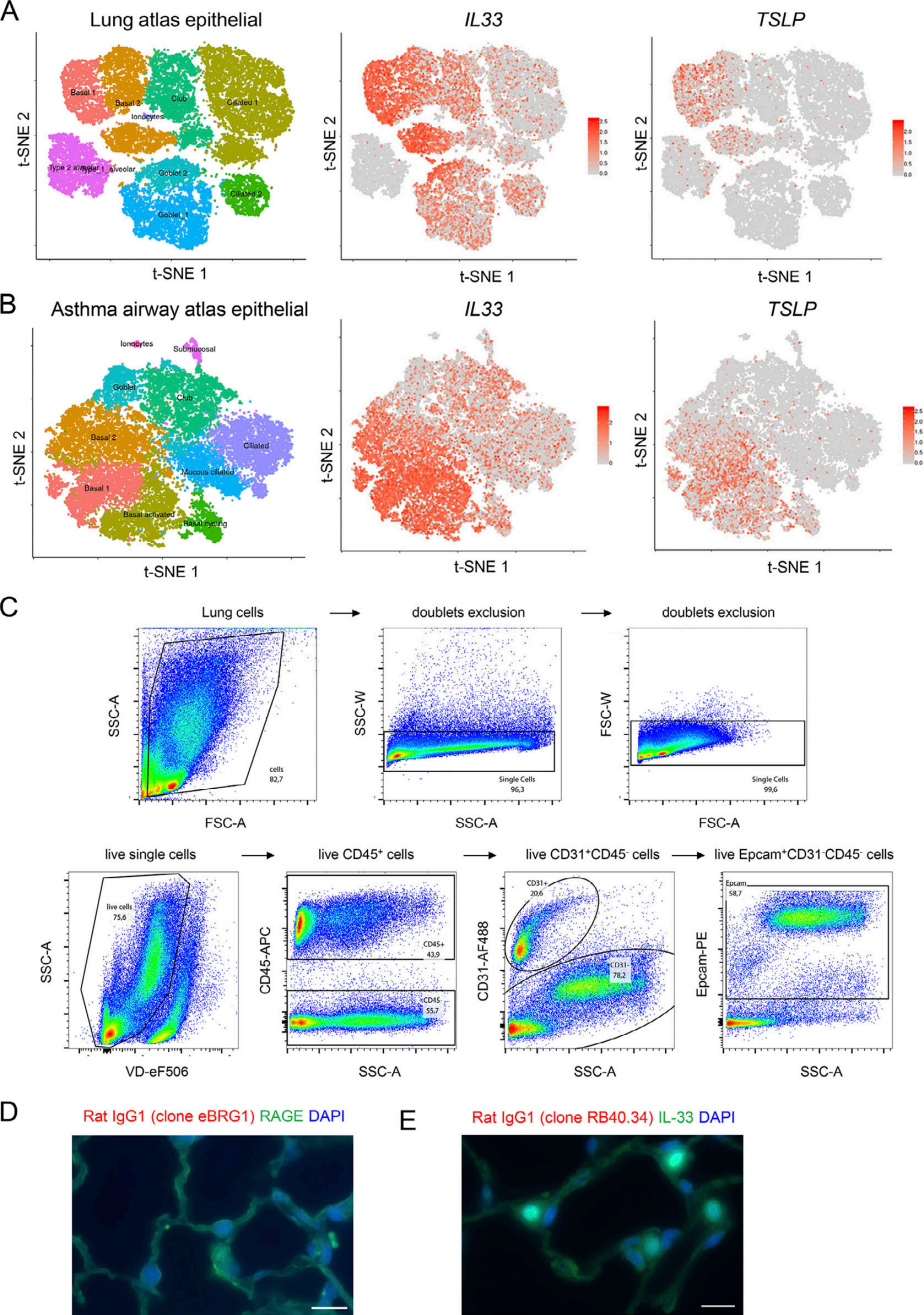
**Single-cell RNA-seq analysis of *IL33* and *TSLP* expression in human lungs and gating strategy for analysis of mouse lung epithelial cells by flow cytometry. (A and B)** Single-cell RNA-seq analysis of *IL33* and *TSLP* expression in epithelial cells from human healthy (A) and asthmatic (B) lungs. t-SNE plots show clustering of 26,154 epithelial cells in upper and lower airways and lung parenchyma in healthy lungs (A; 17 human samples: 6 alveoli and parenchyma, 9 bronchi, 2 nasal), and 25,146 epithelial cells from lower airways in healthy and asthmatic lungs (B; 12 human samples: 15,033 cells from 6 asthma bronchi; 10,113 cells from 6 control bronchi). t-SNE plots were extracted from data obtained by the human lung single-cell atlas (Vieira Braga et al., 2019), and downloaded from https://asthma.cellgeni.sanger.ac.uk. **(C)** Gating strategy of Epcam^+^ epithelial cells and CD31^+^ endothelial cells in the lung of a naïve WT mouse. **(D and E)** Immunohistofluorescence staining of lung tissue sections (naïve wild type C57BL/6J mouse, steady state) with two distinct rat IgG1 isotype controls (rat IgG1 clone eBRG1, D, red; rat IgG1 clone RB40.34, E, red) for the anti-TL1A antibody (rat IgG1, MAB7441, clone 293327). Double staining was performed with antibodies against RAGE (D, green) or IL-33 (E, green). Images are representative of two independent experiments. Scale bar, 10 μm.

We analyzed the expression profile of TL1A in human lungs using datasets from the LungMAP Human Lung CellRef single-cell atlas (Guo et al., 2023). We found that TL1A is constitutively expressed in both type 1 (AT1) and type 2 (AT2) alveolar epithelial cells and basal cells in human healthy lungs (Fig. 1 A). Currently, TL1A is mainly viewed as an inducible cytokine produced by immune cells and endothelial cells under inflammatory conditions (Meylan et al., 2011; Migone et al., 2002; Richard et al., 2015). We thus used independent high-quality datasets to validate our initial observations. We analyzed the Human Cell Atlas (HCA) healthy and asthmatic lung epithelium single-cell RNA-seq datasets (Vieira Braga et al., 2019) and confirmed that TL1A is an epithelial cytokine constitutively expressed in both alveolar epithelium and airway basal cells in healthy human lungs (Fig. 1 B). In addition, we observed that TL1A is expressed in airway basal cells from lower airways in asthmatic lungs (Fig. 1 C). We next used the HCA datasets to compare the expression profile of TL1A with that of epithelial cytokines IL-33, TSLP, and IL-25. IL-33 and TSLP were highly expressed in basal cells from the upper and lower airways in healthy and asthmatic lungs (Fig. S1, A and B). In contrast to IL-33, TSLP, and TL1A, which were easily detected, expression of IL-25 was not detected in any of the lung single-cell RNA-seq datasets that we analysed. We concluded that TL1A is an epithelial cytokine expressed in alveolar epithelium and airway basal cells in human healthy and asthmatic lungs, together with epithelial alarmins IL-33 and TSLP.

### TL1A is expressed in mouse alveolar epithelium at steady state

**Figure 2.**
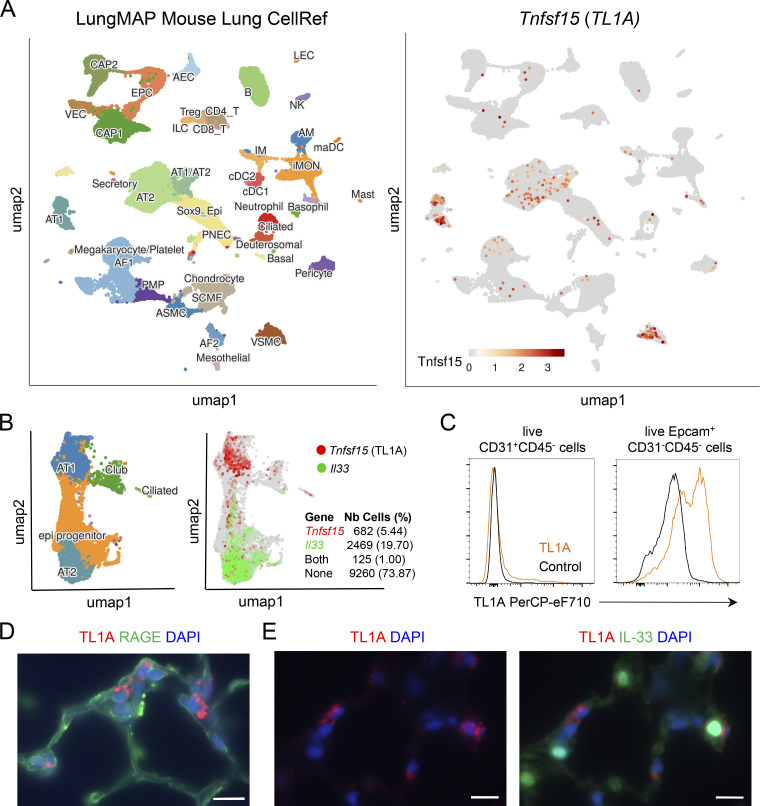
**TL1A is expressed in mouse alveolar epithelium at steady state. (A)** Visualization of *Tnfsf15* (TL1A) expressing cells in the LungMAP single-cell mouse lung atlas. UMAP plots show the clustering of 95,658 lung cells (17 samples from late developmental stage to postnatal day 28). The different cell types in the lungs of naïve mice are indicated on the left. Results are visualized using ShinyCell (Ouyang et al., 2021) and are based upon data generated by the LungMAP Consortium (Guo et al., 2023) and downloaded from http://www.lungmap.net (Gaddis et al., 2024). **(B)** Single-cell RNA-seq analysis of *Tnfsf15/TL1A* and *Il33* gene expression in mouse lung epithelium. UMAP plots show clustering and cell type annotation of 12,536 mouse lung epithelial cells (seven samples from the emergence of the alveolus to postnatal day 28) (Zepp et al., 2021). The number and percentage of epithelial cells expressing *Tnfsf15/TL1A*, *Il33*, or both are indicated on the right. Results are visualized using ShinyCell (Ouyang et al., 2021) and are based upon data obtained by Zepp et al. (2021) and downloaded from http://www.lungmap.net (Gaddis et al., 2024). **(C)** Flow cytometry analysis of cell surface TL1A expression on live CD31^+^CD45^−^ endothelial cells and Epcam^+^CD31^−^CD45^−^ epithelial cells in the lung of a naïve wild type C57BL/6J mouse at steady state. **(D and E)** Immunohistofluorescence staining of lung tissue sections (naïve wild type C57BL/6J mouse, steady state) with antibodies against TL1A (D and E) and RAGE (D) or IL-33 (E) proteins. A tyramide signal amplification (TSA)-based immunofluorescence method was used to detect TL1A-expressing cells in situ. Images are representative of two independent experiments. Scale bar, 10 μm.

We next analyzed TL1A expression in mouse lungs using single-cell RNA-seq datasets from the LungMAP Mouse Lung CellRef single-cell atlas (Guo et al., 2023). We found that, similar to human TL1A, mouse TL1A is constitutively expressed in lung epithelium, in both AT1 and AT2 alveolar epithelial cells (Fig. 2 A). We confirmed these findings using an independent scRNAseq dataset from mouse lung (Zepp et al., 2021). These analyses revealed that while IL-33 is primarily expressed in AT2 cells, TL1A is preferentially expressed in AT1 cells, and only ∼15% of TL1A-expressing cells at baseline co-expressed IL-33 (Fig. 2 B).

We next performed flow cytometry analyses (Fig. S1 C) to study the expression of TL1A protein in the mouse lung at baseline. These experiments revealed the expression of TL1A in Epcam^+^ lung epithelial cells but not in CD31^+^ lung endothelial cells (Fig. 2 C). We then immunostained lung tissue sections with an anti-mouse TL1A mAb (Fig. 2, D and E) or two different rat IgG1 isotype controls (Fig. S1, D and E). We detected expression of endogenous TL1A protein in cells expressing the AT1 cell marker RAGE (Fig. 2 D). TL1A-expressing cells were often located in close proximity to IL-33-expressing cells (Fig. 2 E).

Together, the scRNA-seq, flow cytometry, and immunostaining data indicated that TL1A is an epithelial cytokine constitutively expressed in mouse alveolar epithelium at steady state.

### TL1A synergizes with IL-33 to induce an IL-9-producing ILC9 phenotype in lung ILC2s

**Figure S2.**
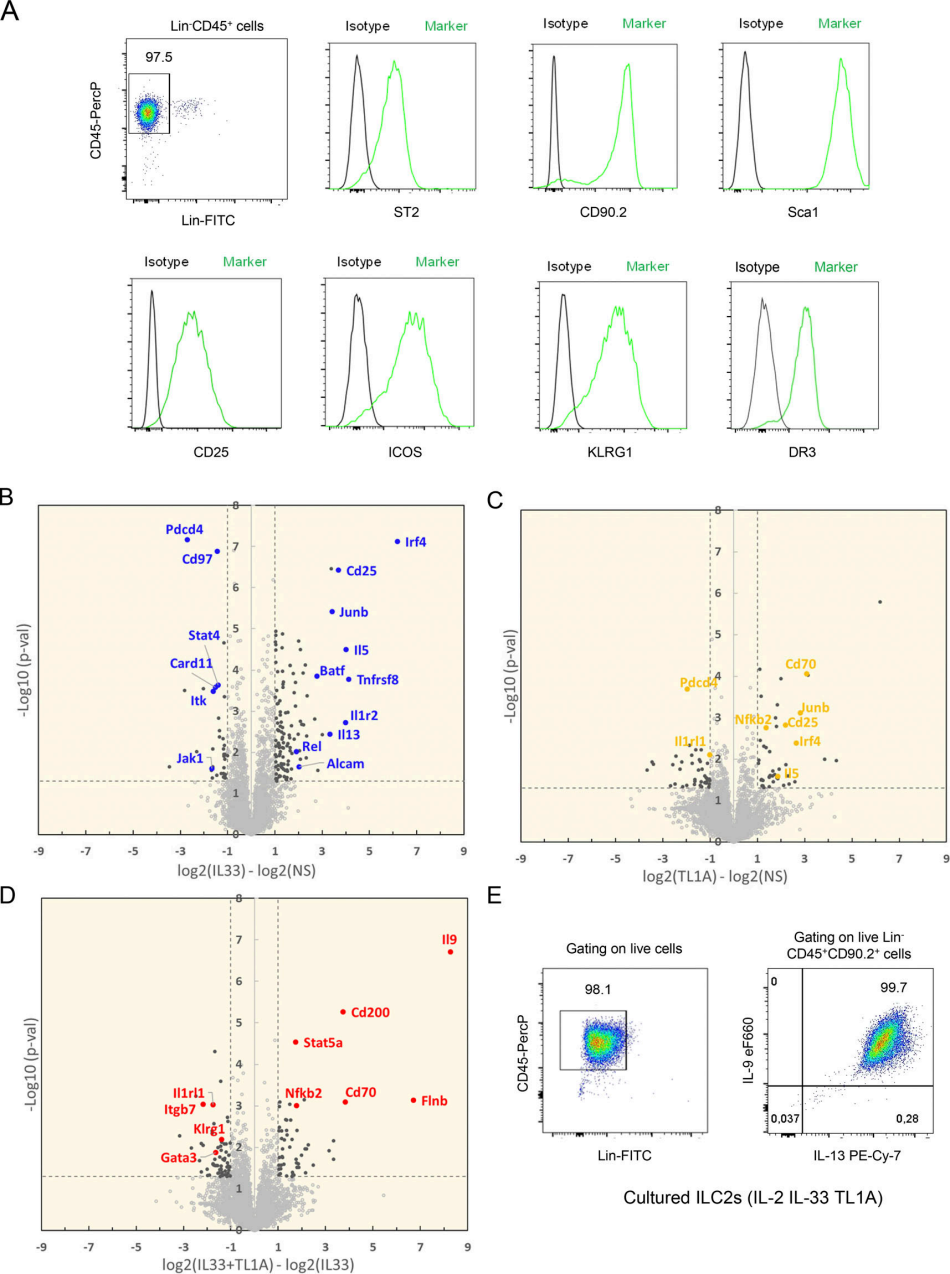
**High throughput proteomic analyses of lung ILC2s stimulated ex vivo with IL-33 and/or TL1A. (A)** Flow cytometry of cultured lung ILC2s ex vivo. Representative histograms of ST2, CD90.2, Sca-1, CD25, ICOS, KLRG1, and DR3 expression at the surface of cultured ILC2s, 3 days after ILC2 cell isolation from the lung and ex vivo culture in the presence of IL-2. Phenotypic analysis was performed on live Lin^–^CD45^+^ cells. **(B–D)** Large-scale label-free proteomic analyses of mouse lung ILC2s after ex vivo overnight stimulation with rIL-2 ± rIL-33 ± rTL1A. Volcano plots of IL-33-stimulated ILC2s (B) or TL1A-stimulated ILC2s (C) compared with non-stimulated cells (NS; in culture with IL-2 alone). Volcano plot of IL-33/TL1A-stimulated ILC2s compared to IL-33-stimulated cells (D). Statistical analysis of protein abundance values was performed from different biological replicate experiments (*n* = 6 for NS and IL33 stimulation; *n* = 3 for TL1A and IL33/TL1A stimulations), using a Student’s *t* test (log_10_ P value, vertical axis). Proteins found as significantly over or under-expressed (P < 0.05 and abs[log_2_ fold change] >1) are shown in black. Representative examples of proteins found modulated in each comparison are shown in color. **(E)** Flow cytometry of cultured lung ILC2s after 14 h of co-stimulation with IL-33 and TL1A in the presence of IL-2 (ILC2 culture used in Fig. 3 C). Intracellular cytokine staining revealed that >99% of ILC2s co-expressed IL-9 and IL-13 intracellularly. Phenotypic analysis was performed on live Lin^−^CD45^+^CD90.2^+^ cells.

**Figure 3.**
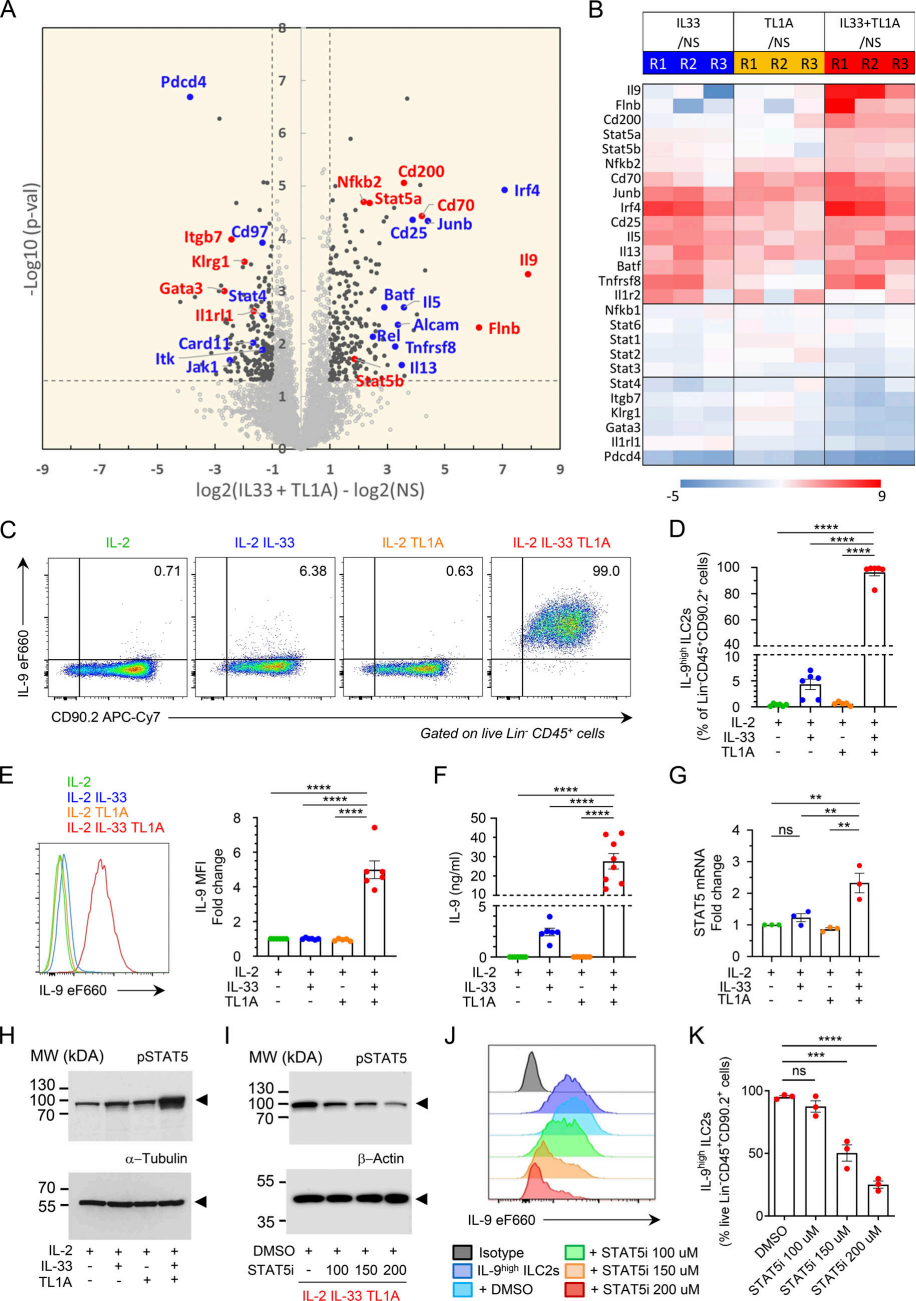
**TL1A synergizes with IL-33 to induce an IL-9-producing ILC9 phenotype in lung ILC2s. (A and B)** Large-scale label-free proteomic analyses of ILC2s isolated from pooled lungs of IL-33-treated *Rag2*^−/−^ C57BL/6 J mice (Schmitt et al., 2018) and cultured with IL-2 (Fig. S2 A) prior to overnight stimulation with rIL-2 ± rIL-33 ± rTL1A. Volcano plot of IL-33/TL1A-stimulated ILC2s (ILC9 cells) compared with nonstimulated cells (NS; in culture with IL-2 alone) (A). Statistical analysis of protein abundance values was performed from different biological replicate experiments (*n* = 6 for NS; *n* = 3 for IL33/TL1A stimulation) using a Student’s *t* test (log_10_ P value, vertical axis). Proteins found as significantly over or under-expressed (P < 0.05 and abs[log_2_ fold change] >1) are shown in black. Examples of proteins modulated in both IL-33/TL1A-stimulated ILC2s and IL-33-stimulated ILC2s are shown in blue. Proteins shown in red are representative of molecules specifically modulated in IL-33/TL1A-stimulated ILC2s (A). Heat-map of fold changes of selected proteins in three independent biological replicates (B). **(C–K)** Analysis of ILC2s isolated from pooled lungs of IL-33-treated *Rag2*^−/−^ C57BL/6 J mice (Schmitt et al., 2018), and cultured with IL-2 prior to 14 h stimulation with rIL-2 ± rIL-33 ± rTL1A. Flow cytometry analysis of live Lin^−^ CD45^+^ cells (C, E, and J), frequency of IL-9^high^ ILC2s (percentage of live Lin^−^ CD45^+^ CD90.2^+^ cells) (D and K), and MFI fold change of IL-9 in ILC2s (E), after cytokines treatment and restimulation by PMA, ionomycin, and brefeldin A (4 h, C–E) or brefeldin A (4 h, J and K). Concentration of IL-9 secreted by ILC2s, measured by ELISA (F). Relative STAT5 mRNA expression levels measured by real-time qPCR (G). Samples were normalized to the expression of HPRT and are shown relative to IL-2-stimulated ILC2s. Immunoblot analysis of activated phosphorylated STAT5 (pSTAT5) and α-tubulin (H) or β-actin (I); Arrowheads indicate the migration of the protein of interest; cropped images. Cultured ILC2s were treated with rIL-2 + rIL-33 + rTL1A and increasing doses of a STAT5 inhibitor (STA5i, CAS 285986-31-4) or control vehicle (DMSO) (I–K). Numbers inside outlined areas (C) indicate percent of cells in the relevant gate. Each symbol represents an individual biological replicate (D–G and K). Data are pooled from six (D and E), six to eight (F) or three (G and K) independent experiments, or are representative of six (C and E) or three (H–J) independent experiments. Data are expressed as mean (±SEM) with P values determined by one-way ANOVA followed by Tukey’s multiple-comparisons test (D–G and K): ns not significant, ** P < 0.01, *** P < 0.001, **** P < 0.0001. Source data are available for this figure: SourceData F3.

**Figure S3.**
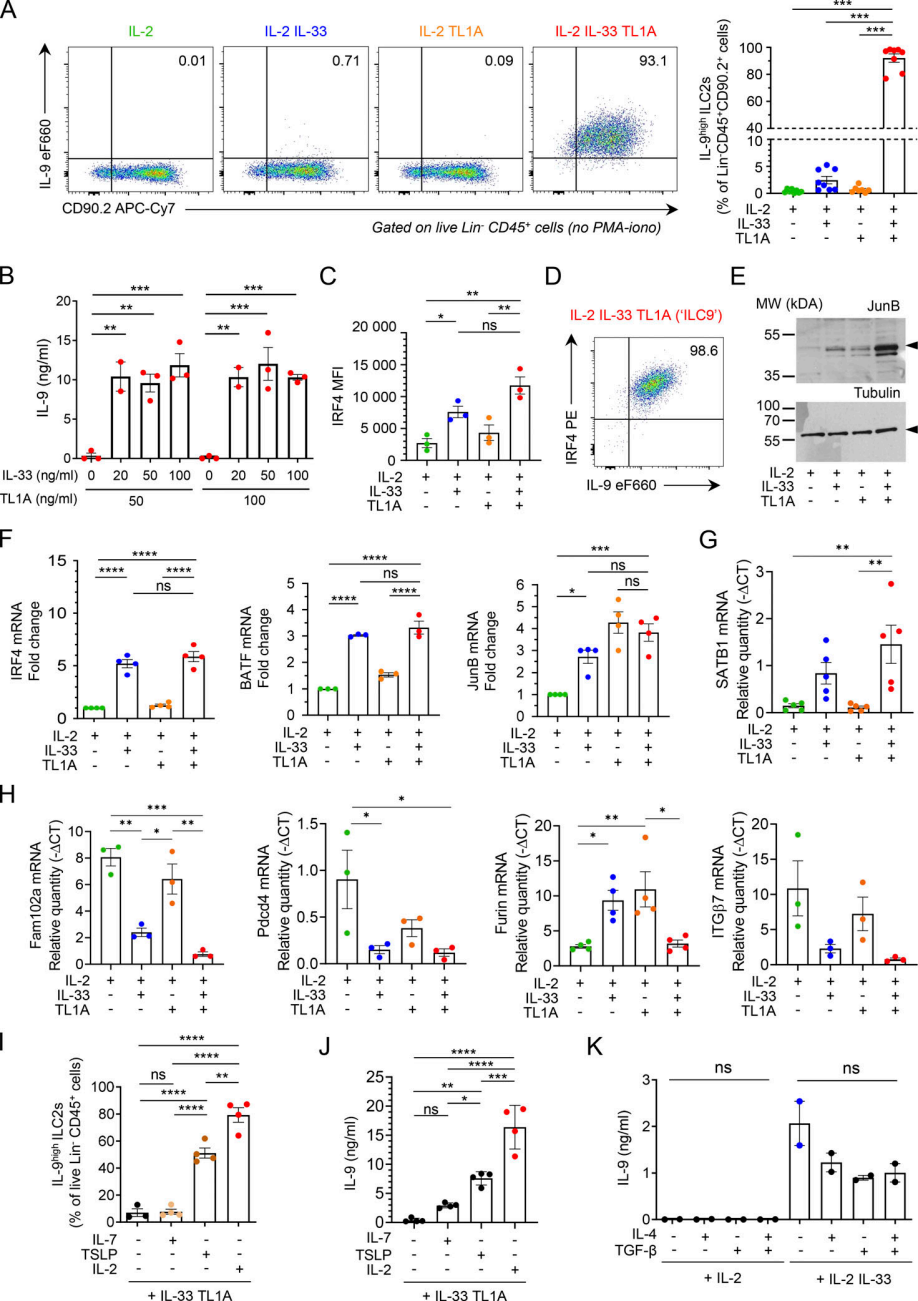
**IL-33 and TL1A synergistically induce IL-9-producing ILC2s ex vivo. (A)** Analysis of cultured lung ILC2s 14 h after ex vivo stimulation by rIL-2 (20 ng/ml) ± rIL-33 (20 ng/ml) ± rTL1A (50 ng/ml). Flow cytometry analysis of live Lin^−^ CD45^+^ cells and frequency of IL-9^high^ ILC2s (percentage of live Lin^−^ CD45^+^ CD90.2^+^ cells) after cytokine treatment and incubation with brefeldin A (4 h), without restimulation by PMA and ionomycin. Numbers inside outlined area indicate percent of cells in the relevant gate and data are representative of eight independent experiments. **(B)** Concentration of IL-9 secreted by ILC2s treated with rIL-2 (20 ng/ml) and various concentrations of rIL-33 and rTL1A measured by ELISA. **(C and D)** MFI of nuclear factor IRF4 (C) and flow cytometry (D) of ILC2s 14 h after ex vivo stimulation of cultured ILC2s by rIL-2 (20 ng/ml) ± rIL-33 (20 ng/ml) ± rTL1A (50 ng/ml). Numbers inside outlined areas (D) indicate percent of cells in the relevant gate and data are representative of three independent experiments. **(E)** Immunoblot analysis of JunB and α-tubulin14 h after cytokine stimulation of lung ILC2s; Arrowheads indicate the migration of the protein of interest; cropped image. Data are representative of three independent experiments. **(F–H)** Relative mRNA expression levels by real time qPCR, 14 h after cytokine stimulation of lung ILC2s. Samples were normalized to the expression of HPRT and data are expressed relative to IL-2-stimulated ILC2s (F) or relative to HPRT mRNA quantity (G and H). **(I and J)** Analysis of mouse lung ILC2s 14 h after ex vivo stimulation by rIL-33 + rTL1A ± rIL-2 ± rIL-7 ± rTSLP. Frequency of IL-9^high^ ILC2s (Lin^−^ CD45^+^ CD90.2^+^ cells), after cytokines treatment and re-stimulation by PMA, ionomycin and brefeldin A (4 h, I). Concentration of IL-9 secreted by ILC2s, measured by ELISA (J). **(K)** Concentration of IL-9 (ELISA) secreted by ILC2s 14 h after ex vivo stimulation by rIL-2 ± rIL-33 ± rIL-4 ± rTGF-β. Each symbol represents an individual biological replicates with *n* = 2–5 independent experiments (A–C and F–K). Data are expressed as mean (±SEM) with P values determined by one-way ANOVA followed by Tukey’s (A, C, and F–J) or Dunnett’s (B and K) multiple-comparisons tests: ns, not significant, * P < 0.05, ** P < 0.01, *** P < 0.001, **** P < 0.0001. In H, all significant P values are annotated with stars, all other comparisons are not significant. Source data are available for this figure: SourceData FS3.

Since we observed constitutive expression of TL1A in barrier epithelial cells, we next investigated its potential cooperation with IL-33 in the early activation of lung ILC2s. We thus set up a large-scale label-free proteomic approach to analyze in an unbiased manner the proteome of ILC2s stimulated with IL-33, TL1A, or IL-33 plus TL1A. Lin^−^CD45^+^ ILC2s isolated from pooled lungs of IL-33-treated *Rag2*^−/−^ C57BL/6 J mice (Schmitt et al., 2018) and cultured with IL-2 expressed the IL-33 receptor ST2, the IL-2 receptor CD25 (IL2Ra), and the TL1A receptor DR3 (encoded by *TNFRSF25*) (Fig. S2 A), in agreement with previous observations (Meylan et al., 2014; Yu et al., 2014). Cultured lung ILC2s were stimulated with IL-2 alone (control, NS) or a combination of IL-2 plus IL-33 (IL-33), IL-2 plus TL1A (TL1A), or IL-2, IL-33 plus TL1A (IL-33+TL1A). We identified and quantified up to 4,438 distinct proteins in control or IL-33-stimulated ILC2s (Table S1). We applied two criteria to derive confident data sets of modulated proteins: Student’s *t* test P value <0.05 and absolute fold change >2. Based on these cut-off values, 159 proteins were found to exhibit a significant variation in the IL-33-activated ILC2 proteome (123 upregulated and 36 downregulated) (Fig. S2 B). ILC2 protein expression was also modulated after treatment with TL1A alone (Fig. S2 C). Although many modulated proteins were present in both the IL-33- and IL-33/TL1A-activated ILC2 proteomes, several proteins were specifically upregulated in the latter (Fig. 3, A and B; and Fig. S2 D). These proteins included signal transducer and activator of transcription factor 5 (STAT5A, STAT5B), transcription factor NF-kB2, filamin B (FlnB), an actin-binding protein that regulates cell migration, and CD200 (Ox-2 membrane glycoprotein), the ligand for CD200R, an inhibitory checkpoint receptor constitutively expressed on lung ILC2s (Shafiei-Jahani et al., 2021). Importantly, our unbiased proteomic analyses revealed that IL-9, a type 2 cytokine associated with allergic inflammation and asthma (Kaplan et al., 2015; Wilhelm et al., 2011, 2012), was the most induced protein in ILC2s costimulated with IL-33 and TL1A (Fig. 3, A and B; and Fig. S2 D). To confirm the results of the high throughput proteomic analyses, we used independent preparations of lung ILC2s cultured with IL-2 prior to ex vivo stimulation with IL-33 and TL1A. Intracellular cytokine staining revealed that up to 99% of ILC2s expressed IL-9 intracellularly after 14 h of costimulation with IL-33 and TL1A (Fig. 3, C and D; and Fig. S2 E), and that IL-9 mean fluorescence intensity (MFI) was highly increased in the costimulated cells (Fig. 3 E). Expression of IL-9 was very strong and detected by intracellular cytokine staining without restimulation of the cells with phorbol 12-myristate 13-acetate (PMA) and ionomycin (Fig. S3 A). Although IL-33 alone induced IL-9 production in cultured ILC2s (1–4 ng/ml; 4 × 10^5^ ILC2s/ml), IL-9 levels were increased 10-fold in supernatants from ILC2s cultured in the presence of IL-33 and TL1A (10–40 ng/ml; 4 × 10^5^ ILC2s/ml) (Fig. 3 F). Treatment with TL1A alone did not induce IL-9 production, even when TL1A was used at a higher dose (Fig. S3 B). Moreover, higher concentrations of IL-33 and TL1A did not further increase IL-9 secretion (Fig. S3 B). We concluded that, upon synergistic activation by IL-33 and TL1A, lung ILC2s acquire an IL-9^high^ phenotype, which we term ILC9 phenotype, characterized by the production of prodigious amounts of IL-9.

Our proteomic analyses revealed the coordinated upregulation in ILC9 cells of IRF4, JunB, and BATF (Fig. 3, A and B), three transcription factors that form immune-specific complexes on AP1-IRF composite elements in regulatory regions of key cytokine genes in T cells (Glasmacher et al., 2012; Li et al., 2012). IRF4, JunB, and BATF bind to the IL-9 promoter and induce IL-9 expression in Th9 cells (Fu et al., 2019; Jabeen et al., 2013; Staudt et al., 2010). IRF4 is also crucial for IL-9 expression in ILC2s (Mohapatra et al., 2016). Flow cytometry analyses confirmed the increase in IRF4 levels in ILC2s stimulated with IL-33 or IL-33/TL1A combination (Fig. S3 C). Up to 98% of ILC2s coexpressed high levels of IL-9 and IRF4 upon costimulation with IL-33 and TL1A (Fig. S3 D). Moreover, Western blot analyses showed a strong increase in the protein levels of JunB (Fig. S3 E). Quantitative PCR (qPCR) analysis indicated that upregulation of IRF4, JunB, and BATF occurred at the mRNA level in ILC9 cells (Fig. S3 F). Indeed, modulation of many proteins identified in the proteomic analyses occurred at the mRNA level (Fig. S3, G and H). Although the upregulation of IRF4, JunB, and BATF by IL-33 and TL1A is likely to be important for IL-9 expression in ILC9 cells, it is not sufficient to explain the synergy between the two cytokines because the three transcription factors were also found in the top 10 modulated proteins in IL-33-activated ILC2s (Fig. 3 B). In contrast, our proteome-wide mass spectrometry analyses (Fig. 3, A and B) and qPCR experiments (Fig. 3 G) revealed that the expression of STAT5, another critical regulator of the *Il9* locus in T cells (Fu et al., 2020), was specifically upregulated at the mRNA and protein level after treatment with IL-33 and TL1A. In T cells, STAT5 functions as a pioneer transcription factor that binds first to the *Il9* promoter and promotes the recruitment of other transcription factors such as BATF (Fu et al., 2020). Thus, the upregulation of STAT5 levels after IL-33/TL1A costimulation could be important for the induction of the ILC9 phenotype. After activation, phosphorylated STAT5 (pSTAT5) proteins dimerize and translocate to the nucleus, where they bind to target genes. We monitored the accumulation of pSTAT5 proteins by Western blot analysis and found that their levels were strongly increased in ILC2s costimulated with IL-33 and TL1A (Fig. 3 H). To determine whether pSTAT5 regulates IL-9 expression in ILC9 cells, we used a specific inhibitor of STAT5 (STAT5i; CAS 285986-31-4) that reduces STAT5 phosphorylation and binding to DNA (Fu et al., 2020; Muller et al., 2008). Treatment with STAT5i reduced the levels of pSTAT5 (Fig. 3 I) and IL-9 production in ILC2s costimulated with IL-33 and TL1A in a dose-dependent manner (Fig. 3, J and K). These later results indicated that pSTAT5 is important for IL-9 expression in ILC9 cells.

IL-2 and TLSP, two known activators of STAT5 phosphorylation, are important for IL-9 production in ILC2s (Mohapatra et al., 2016; Wilhelm et al., 2011). In agreement with these previous studies, we found that IL-2 was required for potent induction of IL-9^high^ ILC2s after costimulation with IL-33 and TL1A ex vivo (Fig. S3 I). Combining TSLP with IL-33 plus TL1A also induced high levels of IL-9 production by lung ILC2s (Fig. S3, I and J). In contrast, IL-7 was not able to rescue massive IL-9 production. We next asked whether TGF-β and IL-4, two cytokines directing T cells to become IL-9-producing cells (Veldhoen et al., 2008), synergize with IL-33 to induce IL-9 production in ILC2s. However, IL-9 secretion was not increased in supernatants from ILC2s cultured with IL-2, IL-33, TGF-β, and IL-4 compared with ILC2s cultured with IL-2 and IL-33 (Fig. S3 K). Thus, TGF-β and IL-4 induce IL-9 production in T cells but not in ILC2s.

**Figure S4.**
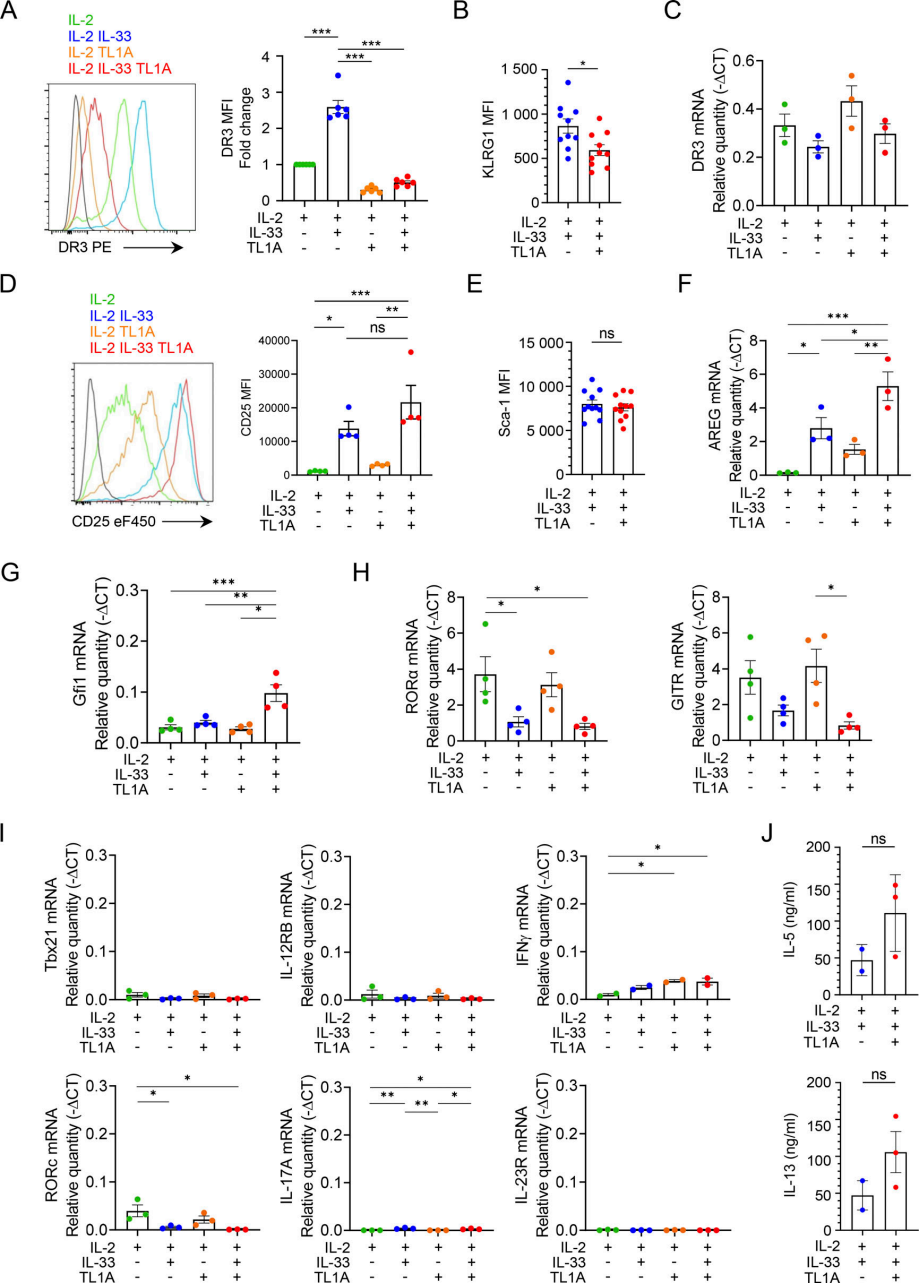
**IL-33 and TL1A induce phenotypic changes in cultured lung ILC2s at the protein and mRNA levels. (A–J)** Analysis of mouse lung ILC2s 14 h after ex vivo stimulation by rIL-2 ± rIL-33 ± rTL1A. MFI of the indicated cell surface markers determined by flow cytometry (A, B, D, and E). Relative mRNA expression levels of various genes (C and F–I), including genes characteristic of ILC1s or ILC3s (I), determined by real-time qPCR, 14 h after cytokine stimulation of lung ILC2s. Samples were normalized to the expression of HPRT and data are expressed as relative to HPRT mRNA quantity. Concentration of IL-5 or IL-13 in cell supernatants, measured by ELISA assay (J). Each symbol represents an individual biological replicate from independent experiments (A–J). Data are expressed as mean (±SEM) with P values determined by unpaired two-tailed Student’s *t* test (B, E, and J) or one-way ANOVA followed by Tukey’s multiple-comparisons test (A, C, D, and F–I): ns, not significant, * P < 0.05, ** P < 0.01, *** P < 0.001. In I, all significant P values are annotated with stars, all other comparisons are not significant.

To further characterize the ILC9 phenotype, we analyzed the expression of cell surface markers associated with ILC2 biology. We noticed that the cell surface levels of the TL1A receptor DR3 and the inhibitory receptor KLRG1 were reduced in ILC2s treated with IL-33/TL1A combination (Fig. S4, A and B). The decrease in DR3 expression was likely due to receptor internalization upon TL1A binding because we did not observe significant changes in DR3 mRNA levels (Fig. S4 C). In contrast to DR3 and KLRG1, expression of the high-affinity IL-2 receptor CD25 was upregulated and that of Sca-1 (Ly6A/E) was not modified (Fig. S4, D and E). In addition, the mRNA levels of tissue-protective factor amphiregulin (*AREG*) and transcription factor *Gfi1* were increased in lung ILC2s exhibiting the ILC9 phenotype (Fig. S4, F and G) and those of RORα and GITR were decreased (Fig. S4 H). Thus ILC9 cells are phenotypically distinct from classical IL-33 activated ILC2s.

### The ILC9 phenotype corresponds to a transient IL-9^high^GATA3^low^ multicytokine producing state of activated ILC2s

**Figure 4.**
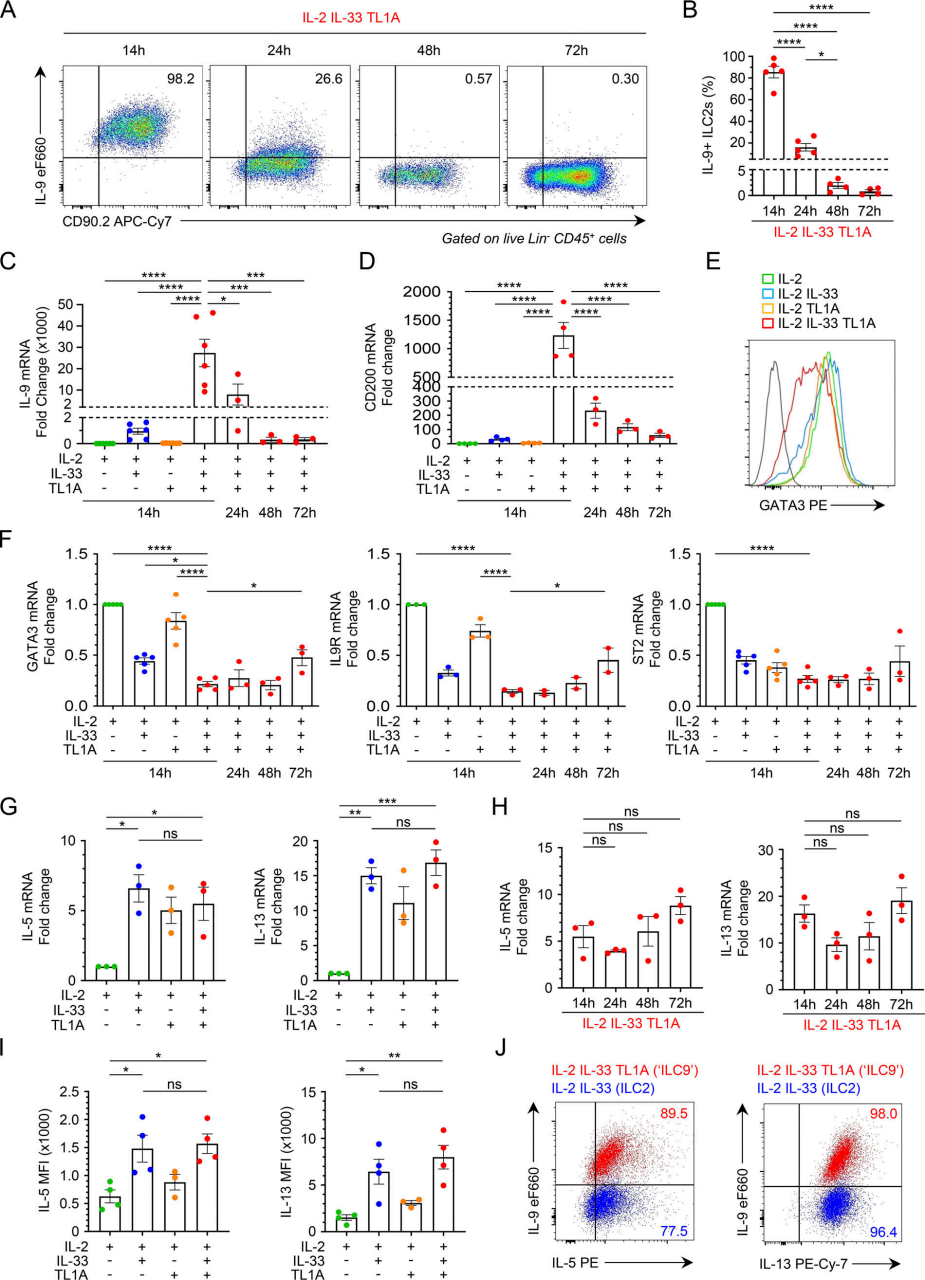
**The ILC9 phenotype corresponds to a transient IL-9**^**high**^**GATA3**^**low**^ **multicytokine producing state of activated ILC2s. (A–J)** Analysis of ILC2s isolated from pooled lungs of IL-33-treated *Rag2*^−/−^ C57BL/6 J mice (Schmitt et al., 2018) and cultured with IL-2 prior to stimulation with rIL-2 ± rIL-33 ± rTL1A. The cells are stimulated for 14 h (E, G, I, and J), except for the kinetic experiments for which the stimulation time is indicated on the graph (A–D, F, and H). Flow cytometry analysis of IL-9, GATA3, IL-5, or IL-13 expression in cultured lung ILC2s (live Lin^−^ CD45^+^ CD90.2^+^ cells) after cytokine treatment and incubation with brefeldin A (4 h), with (A) or without restimulation by PMA and ionomycin (E, I, and J). Numbers inside outlined areas indicate the percent of cells in the relevant gate and data are representative of two to three independent experiments. Frequency of IL-9^high^ ILC2s (Lin^−^ CD45^+^CD90.2^+^ cells) (B), MFI of IL-5 and IL-13 in ILC2s (I), and relative mRNA expression levels of IL-9 (C), CD200 (D), GATA3, IL9R, ST2 (IL1RL1) (F), IL-5 and IL-13 (G and H) by real-time qPCR, 14 h (G and I) or at different time points (B–D, F, and H) after stimulation of lung ILC2s. Samples were normalized to the expression of HPRT and are shown relative to IL-2-stimulated ILC2s (C, D, and F–H). Each symbol (B–D and F–I) represents an individual biological replicate from two to six independent experiments. Data are expressed as mean (±SEM) with P values determined by one-way ANOVA followed by Tukey’s multiple-comparisons test (B–D and F–I): ns, not significant, * P < 0.05, ** P < 0.01, *** P < 0.001, **** P < 0.0001.

We found that the ILC9 phenotype was transiently induced in ILC2s costimulated with IL-33 and TL1A since most ILC2s were IL-9^high^ at 14 h but very few at 48 or 72 h, despite the restimulation with PMA/ionomycin (Fig. 4, A and B). The transient expression of IL-9 protein was caused by the transient production of IL-9 mRNA as shown by qPCR analysis (Fig. 4 C). IL-9 mRNA levels were very high at 14 h (>25,000-fold induction), lower at 24 h, and strongly downregulated after 48 h. Similar to *Il9* mRNA, *CD200* mRNA was transiently induced in ILC2s costimulated with IL-33 and TL1A (Fig. 4 D).

Our proteomic analyses revealed that expression of GATA3, the master transcription factor of ILC2s, was noticeably reduced in cells with an activated ILC9 phenotype (Fig. 3 A). Flow cytometry and qPCR analyses confirmed the downregulation of GATA3 protein (Fig. 4 E) and mRNA (Fig. 4 F) in ILC2s costimulated with IL-33 and TL1A. Lung ILCs constitutively express high levels of *Il9r* mRNA, encoding IL-9R (Price et al., 2010; Wilhelm et al., 2011). In agreement with previous results indicating that *Il9r* is a major target gene of GATA3 (Yagi et al., 2014), *Il9r* mRNA levels were significantly reduced in ILC2s costimulated with IL-33 and TL1A, with kinetics strikingly similar to those of GATA3 mRNA (Fig. 4 F). *Il1rl1,* encoding the IL-33 receptor ST2, is another important target gene of GATA3 in ILC2s (Yagi et al., 2014). Both *Il1rl1* mRNA (Fig. 4 F) and ST2/IL1RL1 protein (Fig. 3 A) were downregulated after costimulation of ILC2s with IL-33 and TL1A. Downregulation of ST2 and DR3 (Fig. S4 A) could contribute, at least in part, to the rapid termination of IL-9 expression in ILC2s stimulated with IL-33 plus TL1A.

Genes characteristic of other ILC lineages, ILC1s (*Ifnγ*, *Il12rb*, *Tbx21*) and ILC3s (*Rorc*, *Il17a*, *Il23r*), were poorly expressed in ILC2s costimulated with IL-33/TL1A, indicating no phenotypic change toward ILC1 or ILC3 phenotypes (Fig. S4 I). Despite the downregulation of GATA3, ILC2s stimulated with IL-33 and TL1A maintained their capacity to produce high levels of IL-5 and IL-13, both at the transcriptional and protein levels (Fig. 4, G–I; and Fig. S4 J). Indeed, intracellular staining revealed that many ILC9 cells coexpressed IL-9 and IL-5 and most of them coexpressed IL-9 and IL-13 (Fig. 4 J). In contrast, IL-33-activated ILC2s expressed IL-5 and IL-13 but only a small subset of these cells (∼2–3%) coexpressed IL-9. Thus, upon synergistic activation by IL-33 and TL1A, lung ILC2s acquire a multicytokine-producing ILC9 phenotype characterized by simultaneous production of large amounts of IL-5, IL-13, and IL-9. We concluded that synergistic activation by IL-33 and TL1A modulates the plasticity of ILC2s and that IL-9 production is associated with a transient phenotypic change toward an IL-9^high^GATA3^low^ activated state.

### TL1A synergizes with IL-33 for induction of IL-9^high^ ILC2s in vivo

**Figure 5.**
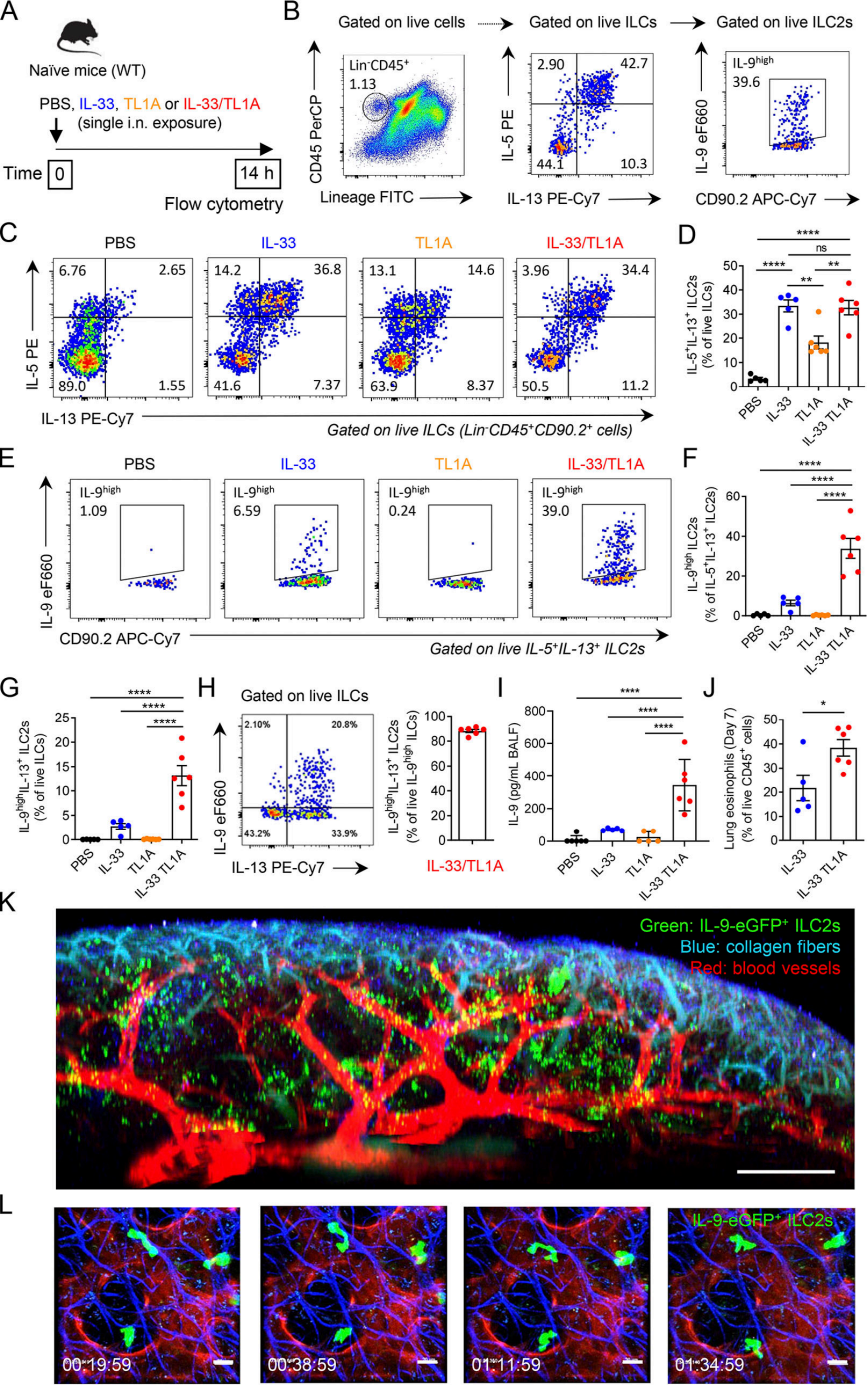
**TL1A cooperates with IL-33 for induction of IL-9**^**high**^ **ILC2s in vivo. (A)** Treatment schedule of naïve wild type (WT, C57BL/6J) mice. **(B)** Gating strategy of IL-9^high^ IL-5^+^IL-13^+^ ILC2s. **(C–I)** Flow cytometry of IL-5^+^IL-13^+^ ILC2s gated on live ILCs (Lin^−^CD45^+^CD90.2^+^ cells) (C) and IL-9^high^ ILC2s gated on live IL-5^+^IL-13^+^ ILC2s (E), frequency of lung IL-5^+^IL-13^+^ ILC2s among live ILCs (D), IL-9^high^ ILC2s among live IL-5^+^IL-13^+^ ILC2s (F), and IL-9^high^IL-13^+^ ILC2s among live ILCs (G) or IL-9^high^ ILCs (H), and concentration of IL-9 in BAL fluids (ELISA assay, I) of WT mice 14 h after a single i.n. administration of PBS or rIL-33 (1 μg) and/or rTL1A (5 μg). Numbers inside outlined areas indicate the percent of cells in the relevant gate and data are representative of two independent experiments (C and E). Each symbol represents an individual mouse and data are pooled from two independent experiments. Data are expressed as mean (±SEM) with P values determined by one-way ANOVA followed by Tukey’s (D) or Dunnett’s (F, G, and I) multiple-comparisons tests: ns, not significant, ** P < 0.01, **** P < 0.0001. **(J)** Frequency of lung eosinophils (Gr1^low^Siglec-F^+^CD11c^−^ cells) among live CD45^+^ cells, at day 7 after a single i.n. exposure to rIL-33 or rIL-33 plus rTL1A. Each symbol represents an individual mouse and data are pooled from two independent experiments. Data are expressed as mean (±SEM) with P values determined by unpaired two-tailed Student’s *t* test: * P < 0.05. **(K and L)** Multiphoton imaging (K) and intravital microscopy (L) of whole lungs of INFER IL-9 fluorescent reporter mice, with detection of IL-9-eGFP^+^ ILC2s (green) and staining of blood vessels (red) and collagen fibers (blue), 16–18 h after a single i.n. administration of IL-33/TL1A combination (1 μg rIL-33 plus 5 μg rTL1A). To increase the numbers of lung IL-9^high^ ILC2s accessible to in vivo imaging, the single i.n. exposure to IL-33/TL1A combination was performed after prior expansion of lung ILC2s by repeated i.p. injections of IL-33 (K and L). Multiphoton image (K) is a 3D reconstitution of stitched images (7 × 7 tiles and 181 z-stack). Time-lapse images (L) illustrate the migratory behavior of IL-9-eGFP^+^ ILC2s. Time in h/min/s. Scale bars: K, 300 μm; L, 20 μm.

**Figure S5.**
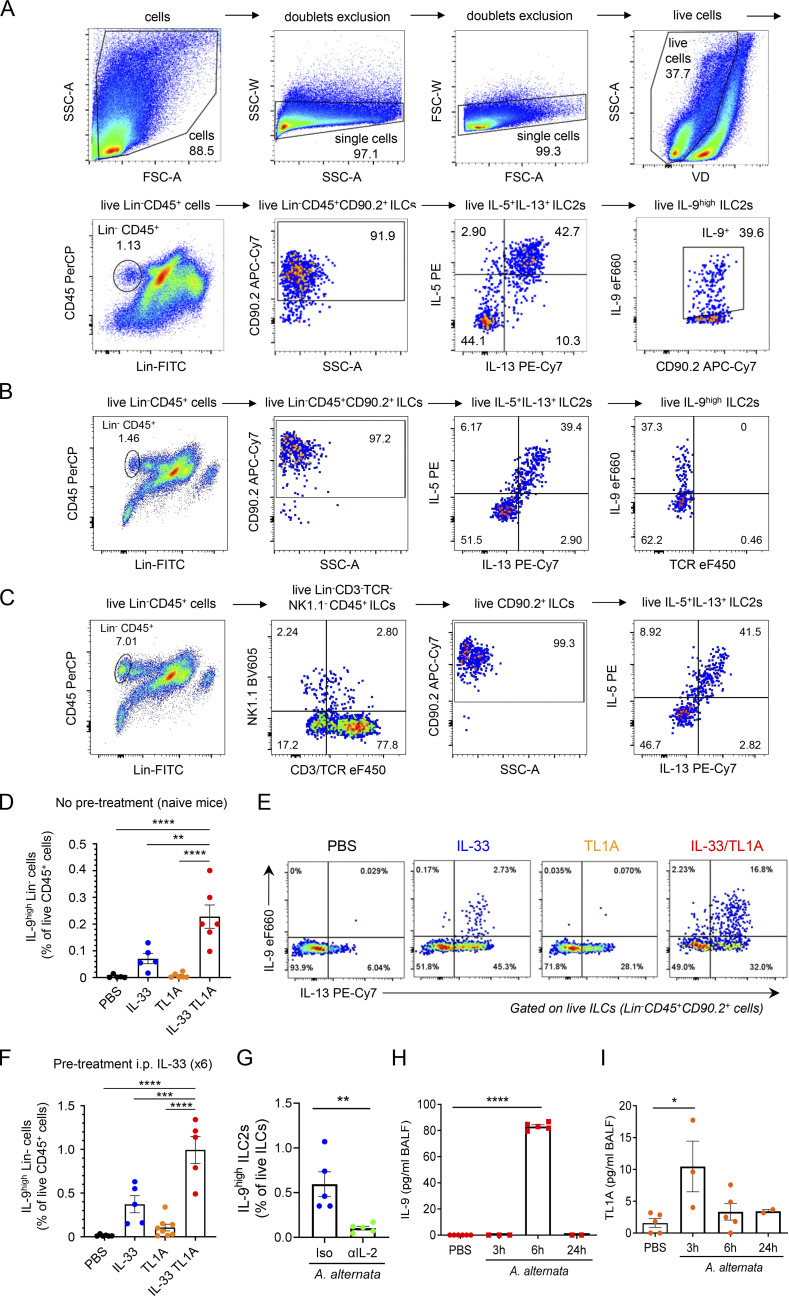
**IL-33 and TL1A synergistically induce IL-9-producing ILC2s in vivo. (A)** Gating strategy and representative flow cytometry plots of live lung ILCs (live Lin^−^CD45^+^CD90.2^+^ cells), live lung IL-5^+^IL-13^+^ ILC2s (live IL-5^+^IL-13^+^ ILCs) and live lung IL-9^high^ ILC2s (live IL-9^high^ IL-5^+^IL-13^+^ ILC2s) in vivo in wild type (WT) C57BL/6J mouse, 14 h after a single i.n. administration of rIL-33 (1 μg) and rTL1A (5 μg). **(B)** Verification of the absence of contamination of the IL-5^+^IL-13^+^ ILC2s and IL-9^high^ ILC2s populations by TCR^+^ cells (T cells and NKT cells) using anti-TCRβ and anti-TCRγδ antibodies. **(C)** Confirmation of the expression of IL-5 and IL-13 in live Lin^−^CD3/TCR^−^NK1.1^−^CD45^+^CD90.2^+^ lung ILCs using antibodies against CD3/TCR and NK1.1 with a different fluorescence from the Lin cocktail (CD4, CD19, CD45R, CD11b, CD11c, Ter119, Ly6G, FcεRI). **(D and E)** Frequency of lung IL-9^high^Lin^−^ cells among live CD45^+^ cells (D), and flow cytometry of IL-9^high^IL-13^+^ ILC2s (live IL-9^high^IL-13^+^Lin^−^CD45^+^CD90.2^+^ cells) (E) of WT mice 14 h after a single i.n. administration of PBS or rIL-33 (1 μg) and/or rTL1A (5 μg). Numbers inside outlined areas indicate the percent of cells in the relevant gate. **(F)** Frequency of lung IL-9^high^Lin^−^ cells among live CD45^+^ cells of WT mice pretreated with six daily i.p. injections of rIL-33 (days 1–6) prior to one i.n. injection of PBS or rIL-33 and/or rTL1A (day 7). Flow cytometry analyses were performed on day 8. **(G)** Frequency of IL-9^high^ ILC2s among live ILCs (Lin^−^CD45^+^CD90.2^+^ cells) in the lungs of WT mice 6 h after a single i.n. administration of *A. alternata* extract (12.5 μg), with (αIL-2 mAb) or without (Iso, isotype control mAb) IL-2 blockade. **(H and I)** Analysis of IL-9 and TL1A release in BAL fluids by ELISA at different time points after the third exposure to *A. alternata* in a chronic exposure model (repeated i.n. administration of 12.5 μg *A. alternata* at days 0, 3, and 6). Each symbol represents an individual mouse and data are pooled from two (D and G) or three (F, H, and I) independent experiments. Data are expressed as mean (±SEM) with P values determined by unpaired two-tailed Student’s *t* tests (G) or one-way ANOVA followed by Dunnett’s multiple-comparison test (D, F, H, and I): * P < 0.05, ** P < 0.01, *** P < 0.001, **** P < 0.0001.

Our proteomic analyses and ex vivo assays were performed with cultured ILC2s. ILC2s isolated from the lungs of IL-33-treated mice and cultured in the presence of IL-2 were no longer naïve ILC2s. To confirm the physiological relevance of our findings, we thus performed in vivo experiments in naïve WT mice. We first determined whether treatment with IL-33 and TL1A was sufficient for the induction of IL-9^high^ ILC2s in vivo. For that purpose, we analyzed IL-5, IL-13, and IL-9 expression in lung ILCs by flow cytometry after a single intranasal (i.n.) exposure of naïve wild type (WT) C57BL/6J mice to IL-33 plus TL1A (Fig. 5 A). Lung ILC2s coproducing IL-5 and IL-13 were identified by gating on live Lin^−^CD45^+^CD90.2^+^ lung ILCs (Fig. 5 B and Fig. S5, A–C). We found that treatment with IL-33, TL1A, or IL-33/TL1A resulted in increased frequencies of IL-5^+^IL-13^+^ ILC2s (Fig. 5 C). We observed similar frequencies after treatment with IL-33 alone and IL-33/TL1A combination indicating no additive effect of the two cytokines (Fig. 5 D). We next analyzed IL-9 expression in IL-5^+^IL-13^+^ ILC2s (Fig. 5 B; and Fig. S5, A and B). A single exposure of naïve WT mice to IL-33, but not to TL1A, was sufficient for the induction of IL-9^high^ ILC2s (Fig. 5 E). However, the responses (∼6% IL-9^high^ ILC2s) were significantly lower than the responses measured with the combination of IL-33 plus TL1A (∼33% IL-9^high^ ILC2s) (Fig. 5, E and F). These results revealed a high level of synergy between IL-33 and TL1A for the induction of IL-9^high^ ILC2s in vivo, in agreement with our in vitro observations with cultured ILC2s. They also showed a specific costimulatory effect of TL1A for induction of IL-9-producing ILC2s by IL-33 in vivo.

We performed additional flow cytometry analyses of lung ILCs without pregating on IL-5^+^IL-13^+^ ILC2s. These analyses revealed that IL-9 expression was restricted to Lin^−^CD45^+^ cells (Fig. S5 D), showing that lung ILCs are the main sources of IL-9 during the initiation of allergic airway inflammation. The vast majority of these IL-9^high^ ILCs coexpressed IL-13 (Fig. 5, G and H; and Fig. S5 E), indicating that they correspond to IL-9^high^ ILC2s. Increased frequencies of IL-9^high^ ILC2s after a single exposure of naïve mice to the combination of IL-33 plus TL1A compared with IL-33 alone (Fig. 5, F and G) was associated with increased levels of IL-9 protein in bronchoalveolar lavage (BAL) fluids at 14 h (Fig. 5 I) and increased frequencies of lung eosinophils (Fig. 5 J), 1 wk after treatment.

We then used multiphoton microscopy and lung intravital microscopy (Lefrancais et al., 2017) to visualize IL-9-producing ILC2s in *IL-9-eGFP* fluorescent reporter mice (Licona-Limon et al., 2013). Multiphoton imaging after lung tissue clearing revealed numerous IL-9-eGFP^+^ ILC2s that accumulated around blood vessels in whole lungs of mice treated with IL-33/TL1A combination (Fig. 5 K, Fig. S5 F, and Video 1). Lung intravital microscopy showed that IL-9-eGFP^+^ ILC2s induced by IL-33/TL1A treatment migrated along collagen fibers and exhibited an “ameboid-like” mode of migration (Fig. 5 L and Video 2), similar to the previously described ameboid-like exploratory movements of activated IL-13-eGFP^+^ ILC2s in the lung peribronchial and perivascular spaces (Puttur et al., 2019). Bright IL-9-eGFP^+^ cells were not present in the lung after i.n. exposure to phosphate buffer saline (PBS) (Video 3). We concluded that IL-33 and TL1A are potent inducers of IL-9-eGFP^+^ ILC2s in the lungs.

**Video 1.** **Related to** Fig. 5 K**.** Endogenous IL-9-producing ILC2s accumulate around blood vessels after IL33/TL1A treatment in vivo. IL9-eGFP^+^ ILC2s (green), blood vessels (Evans Blue/red), and collagen fibers (second harmonic generation/blue) were visualized by multiphoton imaging in the cleared lung of INFER IL9 fluorescent reporter mice 16–18 h after administration of IL33/TL1A combination. 360° rotation of a 3D static representation at a frame rate of 25 fps (500 frames per 20 sec).

**Video 2.** **Related to** Fig. 5 L**.** Endogenous IL-9-producing ILC2s migrate along collagen fibers after IL33/TL1A treatment in vivo. IL9-eGFP^+^ ILC2s (green), blood vessels (Evans Blue/red), and collagen fibers (second harmonic generation/blue) were visualized by lung intravital multiphoton imaging of INFER IL9 fluorescent reporter mice 16–18 h after administration of IL33/TL1A combination. Time in h/min/s. Playback speed: 600.

**Video 3.** **Related to** Fig. 5 L**.** IL-9-producing ILC2s are not present in PBS treated mice. IL9-eGFP^+^ ILC2s (green), blood vessels (Evans Blue/red) and collagen fibers (second harmonic generation/blue) were observed by multiphoton imaging in whole cleared lung of INFER IL9 fluorescent reporter mice 16–18 h after administration of PBS. 360° rotation of a 3D static representation at a frame rate of 25 fps (500 frames per 20 sec).

### Endogenous IL-33 is essential for induction of IL-9^high^ ILC2s during the initiation of allergic airway inflammation

**Figure 6.**
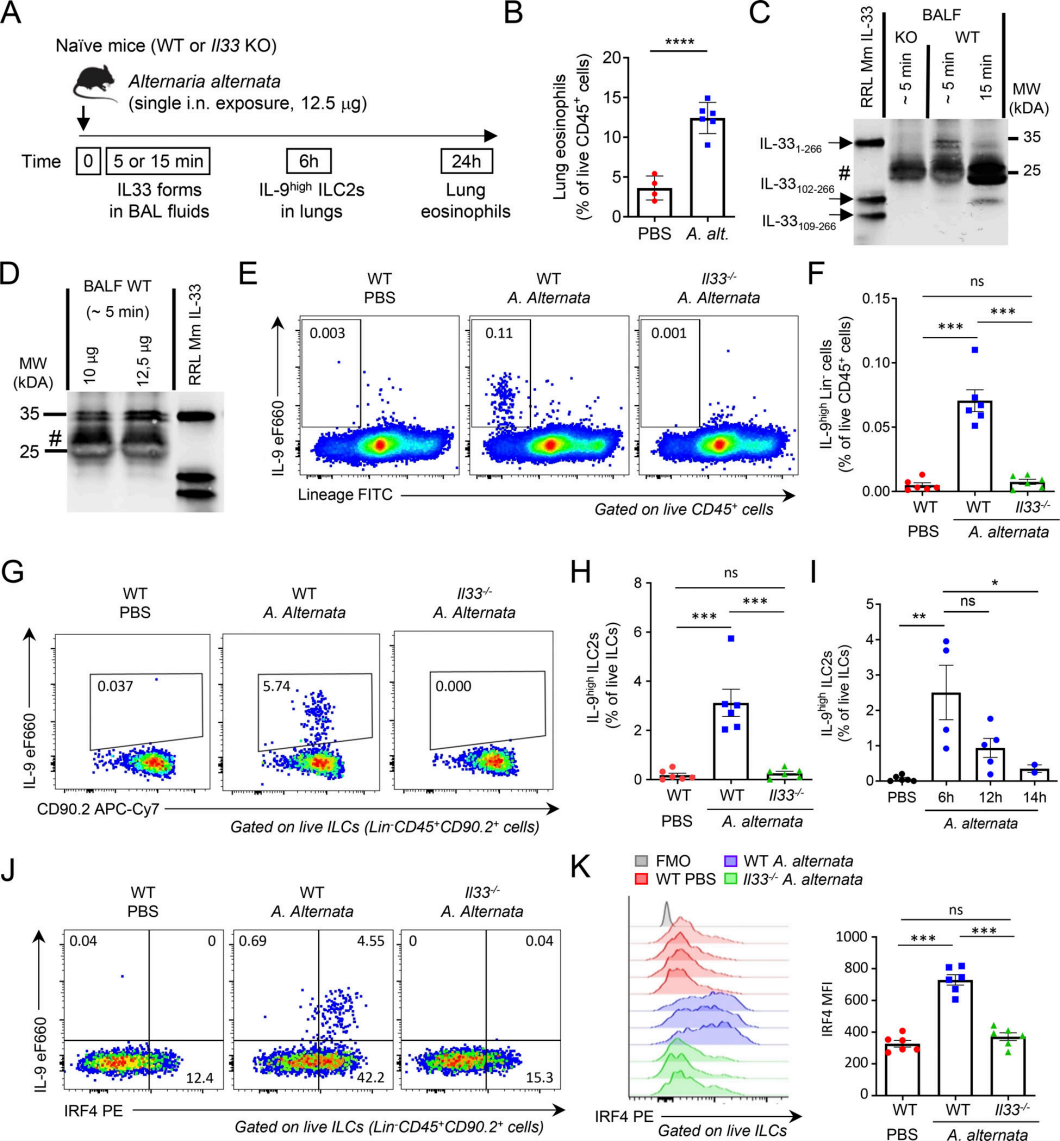
**Endogenous IL-33 is essential for the induction of IL-9**^**high**^ **ILC2s during the initiation of allergic airway inflammation. (A)** Treatment schedule of naïve wild type (WT, C57BL/6J) and *Il33*^*−/−*^ (KO, C57BL/6J) mice. **(B)** Frequency of eosinophils (among live CD45^+^ cells) in the lungs of WT mice 24 h after treatment with a single dose (12.5 μg) of *A. alternata*. **(C and D)** IL-33 forms in BAL fluids from KO mice and WT littermates were analyzed by pull-down assays with ST2-Fc followed by immunoblot with anti-mouse IL-33 antibodies. Recombinant full-length IL-33 (IL-33_1–266_), IL-33_102–266_, and IL-33_109–266_ murine proteins were used as controls in the assays. Naïve mice were exposed to a single i.n. dose of *A. alternata* extracts using different times of exposure (C, 12.5 μg of *A. alternata*) or different amounts of the allergen (D). #, non-specific bands. Blots are representative of two independent experiments. **(E–H)** Flow cytometry and frequency of IL-9^high^Lin^−^ cells among live CD45^+^ cells (E and F) and IL-9^high^ ILC2s among live ILCs (Lin^−^CD45^+^CD90.2^+^ cells) (G and H) in the lungs of WT (Charles River) or *Il33* KO mice 6 h after a single i.n. exposure to *A. alternata*. **(I)** Frequency of IL-9^high^ ILC2s among live ILCs (Lin^−^CD45^+^CD90.2^+^ cells) in the lungs of WT mice at different time points after a single allergen exposure. **(J and K)** Flow cytometry analysis of IL-9 and IRF4 expression in ILC2s (J) and MFI of IRF4 in ILC2s (K) in the lungs of WT (Charles River) or *Il33* KO mice 6 h after a single allergen exposure. Numbers inside outlined areas indicate the percent of cells in the relevant gate (E, G, and J), and data are representative of two independent experiments (mice per group: *n* = 6). Each symbol represents an individual mouse (B, F, H, I, and K) and data are pooled from two (B, F, H, and K) or three (I) independent experiments. Data are expressed as mean (±SEM) with P values determined by unpaired two-tailed Student’s *t* test (B) or one-way ANOVA followed by Tukey’s multiple-comparisons test (F, H, I, and K): ns, not significant, * P < 0.05, ** P < 0.01, *** P < 0.001, **** P < 0.0001. Source data are available for this figure: SourceData F6.

We next analyzed the role of endogenous IL-33 in the induction of IL-9^high^ ILC2s during the onset of allergic inflammation induced by *A. alternata* (Cayrol et al., 2018). A single i.n. exposure of naïve non-sensitized mice to a low dose (12.5 μg) of *A. alternata* extracts (Fig. 6 A) was sufficient for the initiation of allergic airway inflammation, as shown by lung eosinophilia at 24 h after exposure (Fig. 6 B). This treatment resulted in the immediate release (∼5 min) in BAL fluids of endogenous IL-33 protein forms, with an apparent molecular weight similar to that of murine IL-33_FL_ recombinant protein mIL-33_1–266_ (Fig. 6 C). Endogenous IL-33_FL_ proteins migrated as a doublet suggesting post-translational modification. The signal corresponding to IL-33_FL_ proteins was specific since it was not present in BAL fluids from *Il33-*deficient mice exposed to *A. alternata*. Uncleaved IL-33_FL_ forms were also the major forms detected in BAL fluids at early time points after a single exposure to 10 μg of *A. alternata* (Fig. 6 D). Although uncleaved IL-33_FL_ forms were the major forms detected in BAL fluids at early time points (∼5 min), they did not accumulate due to rapid cleavage into a shorter mature form. Indeed, the cleavage product was the major form of IL-33 protein detected in BAL fluids 15 min after allergen exposure (Fig. 6 C). We concluded that endogenous IL-33 protein is released and activated via a two-step mechanism in vivo upon a single exposure to *A. alternata*: immediate release of uncleaved IL-33_FL_ protein in BAL fluids (step 1), followed by rapid proteolytic cleavage into a shorter mature form (step 2). These in vivo results support the model that we previously proposed based on ex vivo observations with human endothelial cells (Cayrol et al., 2018).

We then analyzed phenotypic changes in lung ILC2s shortly (6 h) after IL-33 release and cleavage. We observed a rapid induction of IL-9^high^ Lin^−^CD45^+^ ILC2s (Fig. 6, E and F). This early increase in IL-9^high^ ILC2s upon a single i.n. exposure of naïve mice to *A. alternata* extracts was not observed in *Il33*^*−/−*^ mice. Importantly, no IL-9^high^ Lin^+^CD45^+^ cells (including CD3^+^ and CD4^+^ T cells) were found at this early time point (6 h), indicating that Lin^−^CD45^+^ ILC2s were the only cells producing IL-9 shortly after allergen exposure and IL-33 release. IL-9^high^ ILC2s represented up to 5% of lung ILCs after a single i.n. exposure to *A. alternata* (Fig. 6, G and H). We observed higher frequencies of IL-9^high^ ILC2s at 6 h than at 14 h (Fig. 6 I). We next analyzed the expression of IRF4 and detected a strong induction in lung ILC2s 6 h after the single allergen exposure (Fig. 6, J and K). The upregulation of IRF4 and the induction of IL-9^high^ IRF4^high^ ILC2s were strictly dependent on the expression of IL-33. We concluded that endogenous IL-33 protein plays a crucial role in the rapid induction of IL-9^high^ ILC2s during the initiation of allergic airway inflammation.

Since IL-2 was important for the induction of the ILC9 phenotype ex vivo, we tested the effects of IL-2 neutralization and found that this treatment significantly reduced the frequency of IL-9^high^ ILC2s 6 h after exposure of naïve WT mice to *A. alternata* (Fig. S5 G). Thus, endogenous IL-2 is required, in addition to endogenous IL-33, for rapid induction of IL-9^high^ ILC2s after a single allergen exposure.

### Endogenous TL1A functions as an epithelial alarmin important for early induction of IL-9^high^ ILC2s after allergen exposure

**Figure 7.**
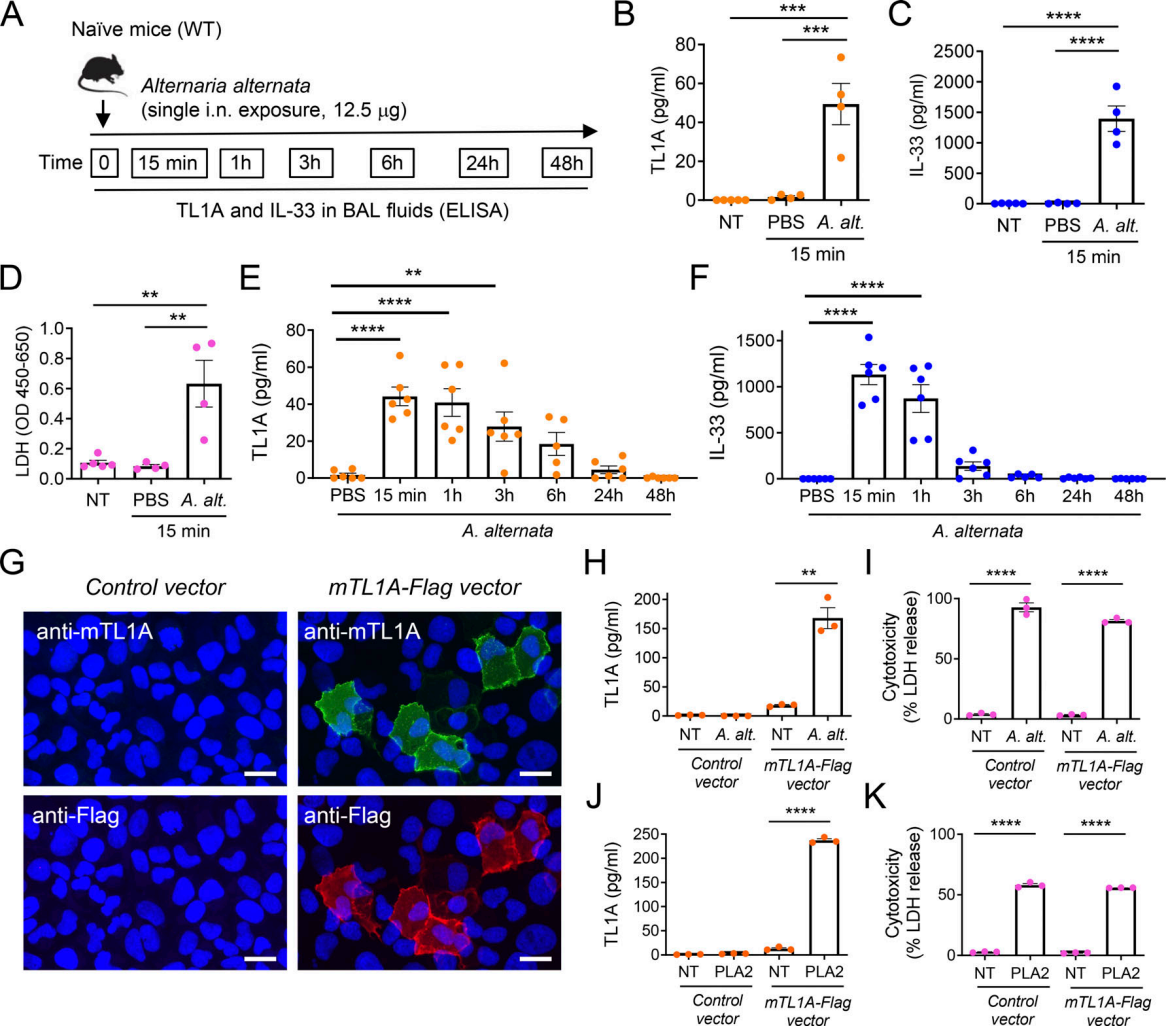
**Endogenous TL1A functions as an epithelial alarmin rapidly released after allergen exposure. (A)** Treatment schedule of naïve wild type (WT, C57BL/6J) mice. **(B–F)** Analysis of TL1A and IL-33 release in BAL fluids after a single allergen exposure. TL1A (B and E), IL-33 (C and F), and LDH (D) levels in BAL fluids were determined by ELISA (B, C, E, and F) or LDH (D) assays, 15 min (B–D) or at different time points (E and F) after a single i.n. administration of *A. alternata* extract (12.5 μg). Each symbol represents an individual mouse and data are pooled from two independent experiments (B–F). Data are expressed as mean (±SEM) with P values determined by one-way ANOVA followed by Tukey’s (B–D) or Dunnett’s (E and F) multiple-comparisons tests: ** P < 0.01, *** P < 0.001, **** P < 0.0001. **(G–K)** Analysis of TL1A release in cell supernatants after exposure of TL1A-expressing cells to *A. alternata* or bee venom phospholipase A2 (PLA2). U2OS epithelial cells transfected with a mouse TL1A-Flag expression vector (mTL1A-Flag vector) or control vector were analyzed by indirect immunofluorescence microscopy with anti-mTL1A and anti-Flag antibodies (G). Scale bar, 20 μm. TL1A (H and J) and LDH (I and K) levels in cell supernatants were determined by ELISA (H and J) or LDH cytotoxicity assays (I and K) 15 min after treatment with *A. alternata* extract (*A. alternata*, H and I) or 1 h after treatment with bee venom PLA2 (J and K). NT, not treated. Each symbol represents an individual biological replicate and data are pooled from three independent experiments (H–K). Data are expressed as mean (±SEM) with P values determined by unpaired two-tailed Student’s *t* tests (treatment versus NT): ** P < 0.01, **** P < 0.0001.

We next investigated the role of endogenous TL1A in the induction of IL-9^high^ ILC2s during the onset of allergic inflammation. Since TL1A is constitutively expressed in lung epithelium at baseline, we asked whether it functions as an alarmin. We found that a single i.n. treatment of naïve mice with *A. alternata* extracts (Fig. 7 A) resulted in the release of TL1A in BAL fluids within the first 15 min after allergen exposure (Fig. 7 B), similar to IL-33, the prototypical epithelial alarmin (Fig. 7 C). Exposure to *A. alternata* is known to cause damage to barrier epithelial cells (Scott et al., 2018). Accordingly, we observed that release of TL1A and IL-33 was associated with increased levels of LDH, a marker of membrane damage and cell death, in BAL fluids 15 min after a single exposure to *A. alternata* (Fig. 7 D). Kinetic analyses over a 48 h period after allergen exposure (Fig. 7 A) revealed that TL1A accumulated in BAL fluids during the first hours and declined at later time points (24–48 h) (Fig. 7 E). IL-33 also accumulated during the first hour and declined very rapidly (Fig. 7 F). Together, these results supported the mode of action of TL1A as an epithelial alarmin: TL1A is constitutively expressed in lung epithelial cells at baseline (preformed) and rapidly (and transiently) released in BAL fluids upon cell damage caused by a single exposure to *A. alternata*.

To confirm this mechanism using an independent approach, we ectopically expressed mouse TL1A (mTL1A) in an epithelial cell line (Fig. 7 G). We detected expression of epitope-tagged TL1A by indirect immunofluorescence staining in cells transfected with mTL1A-Flag expression vector, but not in cells transfected with control vector. Exposure of cells expressing mTL1A to *A. alternata* resulted in the rapid release of TL1A in supernatants 15 min after allergen treatment (Fig. 7 H). TL1A release correlated with LDH release at this early time point (Fig. 7 I). In contrast, TL1A and LDH were not released from transfected cells in the absence of treatment (NT). *A. alternata* also induced LDH release in cells transfected with the control vector, but TL1A was not detected in supernatants, as expected. Finally, we observed TL1A and LDH release after exposure of cells expressing mTL1A to bee venom phospholipase A2 (PLA2) (Fig. 7, J and K), a potent allergen that causes cell damage and induces IL-33 release in vitro and in vivo (Cayrol et al., 2018). Thus, TL1A functions as an alarmin that is rapidly released upon cellular damage.

**Figure 8.**
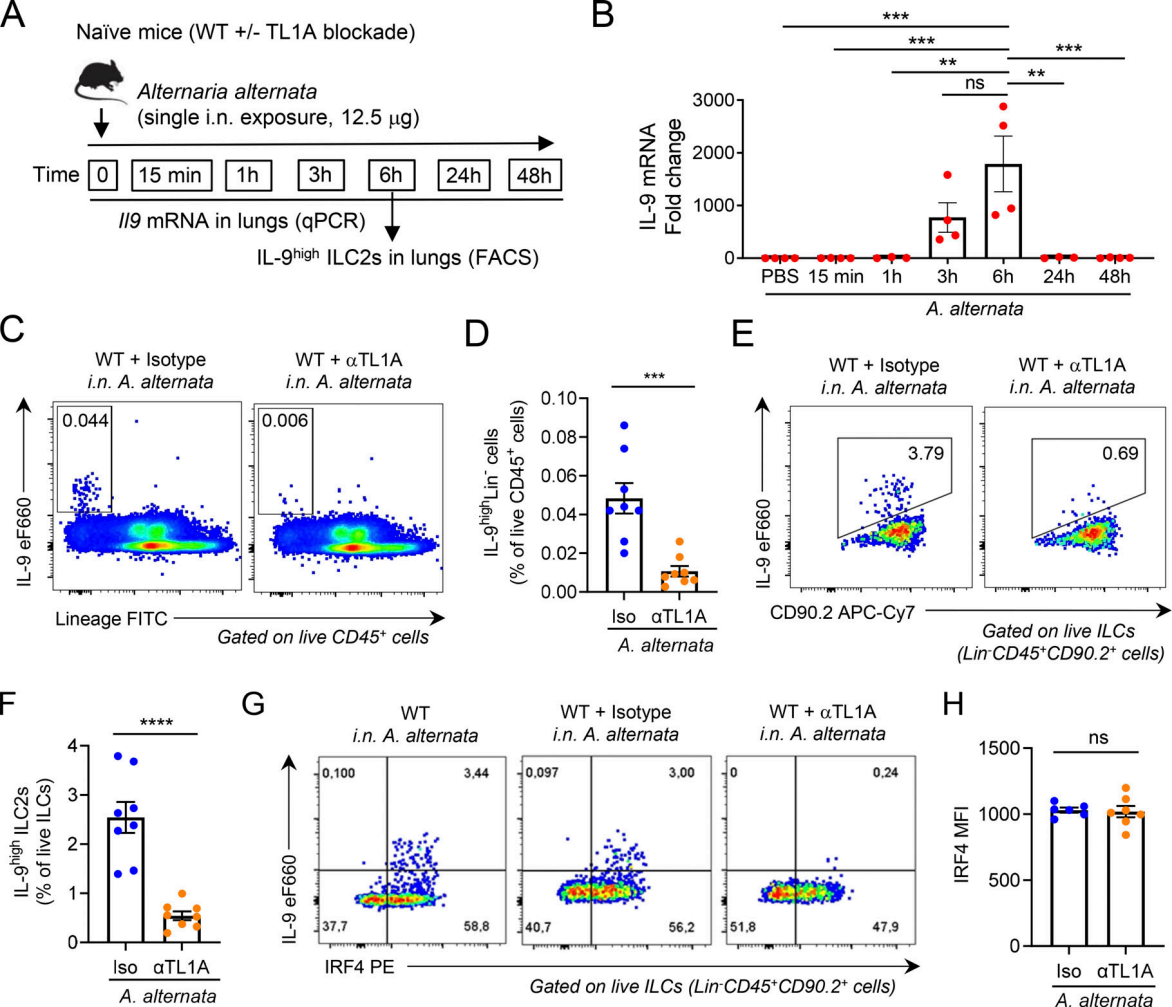
**Endogenous TL1A is important for early induction of IL-9**^**high**^ **ILC2s after allergen exposure. (A)** Treatment schedule of naïve WT mice. **(B)** IL-9 mRNA levels in the lungs analyzed by qPCR at different time points after a single allergen exposure. Data are expressed as relative to IL-9 mRNA levels in mice treated with PBS. **(C–H)** Flow cytometry and frequency of IL-9^high^Lin^−^ cells among live CD45^+^ cells (C and D) and IL-9^high^ ILC2s among live ILCs (Lin^−^CD45^+^CD90.2^+^ cells) (E and F), flow cytometry (G), and MFI of IRF4 expression in ILC2s (H), in the lungs of WT mice 6 h after a single i.n. administration of *A. alternata* extract (12.5 μg), with (αTL1A mAb) or without (Iso, isotype control mAb) TL1A blockade. Numbers inside outlined areas indicate the percent of cells in the relevant gate (C, E, and G) and data are representative of two (G) or three (C and E) independent experiments. Each symbol represents an individual mouse and data are pooled from three (D and F) or two (B and H) independent experiments. Data are expressed as mean (±SEM) with P values determined by one-way ANOVA followed by Tukey’s multiple-comparisons test (B) or unpaired two-tailed Student’s *t* tests (D, F, and H): ns, not significant, *** P < 0.001, **** P < 0.0001.

We next analyzed IL-9 mRNA induction in the lungs after a single exposure of naïve WT mice to *A. alternata* (Fig. 8 A). We found that IL-9 mRNA was strongly induced a few hours after IL-33 and TL1A release (Fig. 8 B). The upregulation of IL-9 mRNA was transient. It was maximum at 6 h and no longer observed 24 and 48 h after allergen exposure. We observed a similar transient induction of IL-9 production 6 h after the last exposure to *A. alternata* in a context of chronic exposure, i.e., three repeated i.n. treatments with the allergen over a 1-wk period (Fig. S5 H). Similar to the results obtained in the single allergen exposure experiments, TL1A was released in BAL fluids a few hours before IL-9 production (Fig. S5 I).

We then determined the impact of a TL1A function-blocking antibody on the induction of IL-9^high^ ILC2s after a single exposure of naïve WT mice to *A. alternata* and found that this treatment strongly reduced the frequency of IL-9^high^ ILC2s at 6 h (Fig. 8, C to F). In contrast to *Il33* deletion, TL1A blockade did not modify the expression of IRF4 in lung ILC2s (Fig. 8, G and H). Thus, endogenous TL1A regulates the induction of IL-9^high^ ILC2s, independently of IRF4 expression. We concluded that TL1A is an epithelial alarmin constitutively expressed in lung epithelium, rapidly released in BAL fluids after allergen exposure, and important for early induction of IL-9 production by lung ILC2s.

### ILC9 cells have an increased capacity to initiate IL-5-dependent allergic airway inflammation

**Figure 9.**
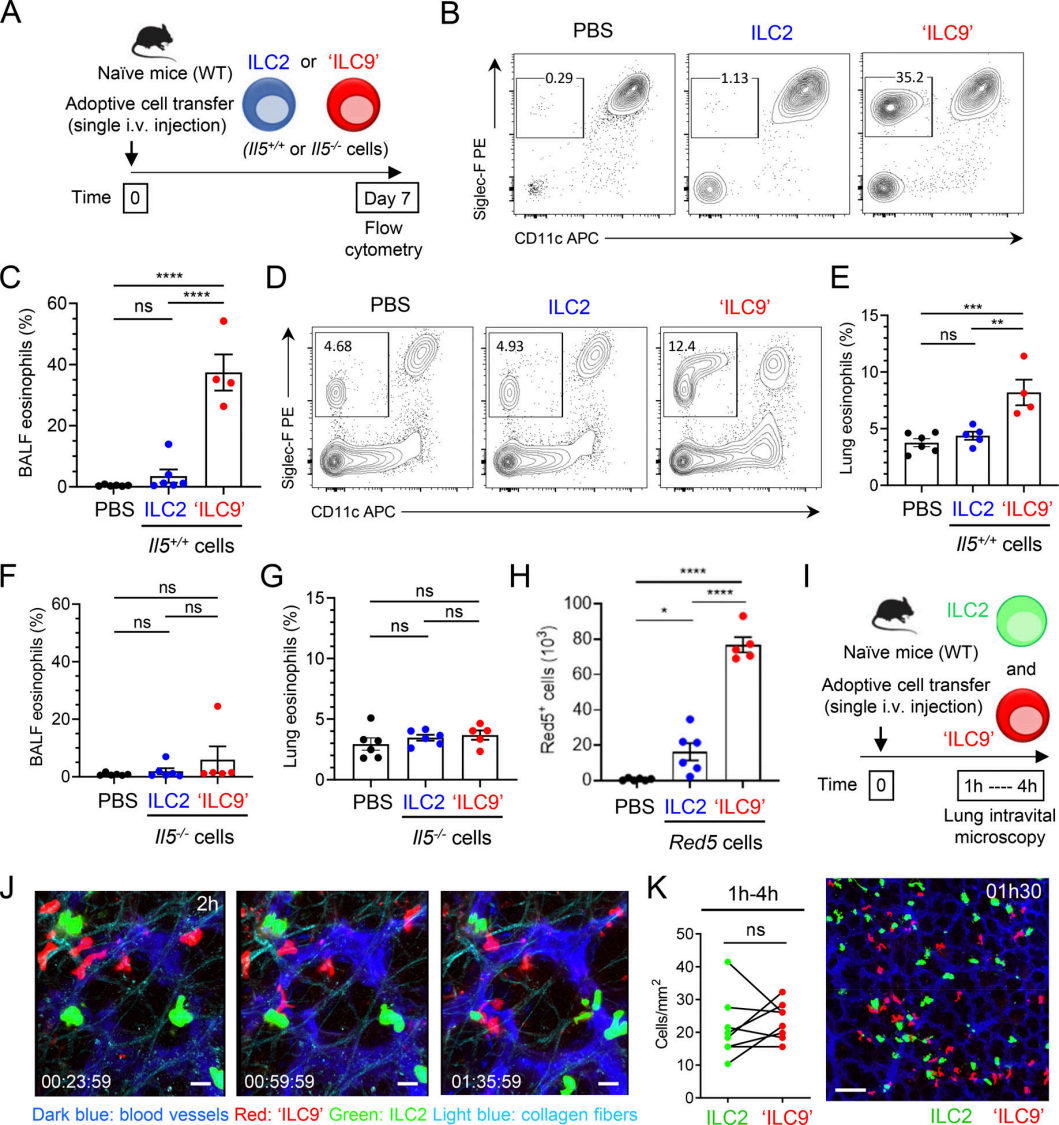
**ILC9 cells have an increased capacity to initiate IL-5-dependent allergic airway inflammation. (A)** Treatment schedule of naïve wild type (WT, C57BL/6J) mice by a single i.v. adoptive cell transfer of classical IL-33-activated ILC2s (ILC2) or IL-33/TL1A-activated ILC2s (ILC9). **(B–H)** Flow cytometry (B and D) and frequency of eosinophils (Gr1^low^Siglec-F^+^CD11c^−^ cells) among live CD45^+^ cells from BALF (C and F) or lung (E and G), and number of Red5^+^ ILC2s or ILC9s in total lung of mice (H), at day 7 after a single i.v. adoptive transfer of 5 × 10^5^ ILC2s or ILC9s in separate host mice. Adoptively transferred ILC2s and ILC9s were prepared from *Rag2*^−/−^ mice (*Il5*^*+/+*^ cells) (B–E) or *Red5* mice (*Il5*^*−/−*^ cells) (F–H). Control mice received an intravenous injection of PBS. Red5^+^ cells indicate the activity of the *Il5* promoter. Each symbol represents an individual mouse and data are representative (B and D) or pooled (C and E–H) from two independent experiments. **(I–K)** Live imaging of ILC2s and ILC9 cells in the lung. Lung intravital microscopy was performed 1–4 h after adoptive transfer of 6 × 10^5^ of each cell type in the same host (green, classical IL-33-activated ILC2s-CFSE^+^; red, IL-33/TL1A-activated ILC9 cells-CTO^+^) (I). Imaging of the migratory behavior of ILC2s and ILC9 cells in the lung (J) and cell quantification from lung intravital microscopy data (K). Time-lapse images, 2 h after adoptive cell transfer (J). A maximum intensity projection of stitched images (2 × 2 tiles and 18 z-stack) is shown (K). Time in h/min/s. Scale bars: J, 20 μm; K, 100 μm. Lung intravital microscopy data are representative (J and K) or analyzed (K) from three adoptive transfer experiments on four mice. Data are expressed as mean (±SEM) with P values determined by paired two-tailed Student’s *t* test (K) or one-way ANOVA followed by Tukey’s multiple-comparisons test (C and E–H): ns, not significant, * P < 0.05, ** P < 0.01, *** P < 0.001, **** P < 0.0001.

We next developed an adoptive cell transfer approach to analyze the impact of ILC2s exhibiting the ILC9 phenotype on allergic airway inflammation in vivo. Lung ILC2s were stimulated ex vivo with IL-33 (classical IL-33-activated ILC2s) or IL-33/TL1A combination (ILC9 cells) and adoptively transferred into naïve WT mice (Fig. 9 A). We observed increased numbers of eosinophils in BAL fluids (Fig. 9, B and C) and lungs (Fig. 9, D and E), 7 days after a single i.v. injection of 5 × 10^5^ ILC9 cells. In contrast, BAL fluid and lung eosinophilia were not observed 7 days after a single i.v. transfer of the same number of classical IL-33-activated ILC2s (Fig. 9, B–E). Adoptive transfer experiments with cells isolated from homozygous *Red5* mice (*Il5*^*−/−*^ mice), instead of *Il5*^*+/+*^ mice (Fig. 9 A), revealed that the effects of ILC2s exhibiting the ILC9 phenotype on airway eosinophilia were entirely dependent on their own production of IL-5 (Fig. 9, F and G). ILC2s from *Red5* mice express the red fluorescent tdTomato protein (Red5^+^ cells), when the IL-5 promoter is activated (Nussbaum et al., 2013). We detected higher numbers of Red5^+^-activated ILC9 cells, than Red5^+^-activated ILC2s, in the lungs 7 days after a single adoptive transfer of 5 × 10^5^ cells (Fig. 9 H). We concluded that ILC9 cells have an increased capacity to persist in vivo in an activated IL-5-producing state and to initiate IL-5-dependent allergic airway inflammation.

We performed additional experiments to detect potential differences in lung-homing and recruitment properties of the cells. We intravenously coinjected ILC2s exhibiting the activated ILC2 (green) or ILC9 (red) phenotypes in the same host and analyzed their behavior by lung intravital microscopy (Fig. 9 I). These analyses revealed that both populations entered the lungs during the first hours after transfer and were highly dynamic (Fig. 9 J and Video 4). Similar to the endogenous IL-9-eGFP^+^ ILC2s induced by IL-33/TL1A treatment (Fig. 5 L and Video 2), the adoptively transferred ILC9 cells exhibited an ameboid-like mode of migration (Video 4). We frequently observed conversion from usual round shape of ILC2s to spindle-shaped morphology, a characteristic of migratory and invasive cells. Quantification of fluorescent cells in the lungs did not reveal a significant difference between the numbers of IL-33-activated ILC2s and ILC9 cells that accumulated during the first hours after transfer (Fig. 9 K). We concluded that the higher numbers of ILC9 cells found in the lungs 7 days after adoptive transfer, compared to classical IL-33-activated ILC2s, are unlikely to be due to differences in lung-homing properties of the cells.

**Video 4.** **Related to** Fig. 9 J**.** Adoptively transferred ILC2s and ILC9s are equally recruited to the lung and exhibit an ameboid-like mode of migration. IL-33-activated ILC2s (CFSE/green), IL33/TL1A-activated ILC9s (CTO/red), blood vessels (Evans Blue/dark blue), and collagen fibers (second harmonic generation/light blue) were observed by lung intravital multiphoton imaging 2 h after intravenous adoptive transfer (6 × 10^5^ cells). Time in h/min/s. Playback speed: 600.

## Discussion

In the present study, we demonstrate that TL1A is an epithelial alarmin, constitutively expressed in airway basal cells and alveolar epithelium at steady state in both mice and humans, which cooperates with IL-33 for induction of IL-9^high^ ILC2s during the onset of allergic airway inflammation. The synergistic effect of IL-33 and TL1A in the induction of IL-9 synthesis by lung ILC2s is particularly striking with up to 98% of the cells exhibiting an IL-9^high^ ILC9 phenotype after costimulation ex vivo. We show that a single treatment with IL-33 and TL1A is sufficient for induction of IL-9-producing ILC2s in vivo and that endogenous IL-33 and TL1A are necessary for rapid induction of IL-9^high^ ILC2s after a single exposure of naïve mice to *A. alternata*, a major asthma-associated allergen. Large-scale proteomics and kinetic analyses indicated that ILC9 cells are phenotypically distinct from classical IL-33 activated ILC2s, and that the ILC9 phenotype corresponds to a transient IL-9^high^GATA3^low^ multicytokine producing state of activated ILC2s, characterized by simultaneous production of large amounts of type 2 cytokines IL-9, IL-5, and IL-13, and downregulation of GATA3 and its target genes *Il9r* and *Il1rl1*. Adoptive transfer experiments revealed that ILC9 cells have an increased capacity to persist in an activated IL-5-producing state in vivo and to initiate IL-5-dependent allergic airway inflammation. We thus believe that an important role of TL1A is to increase the amplitude and duration of the IL-33 alarm signal through the synergistic induction of IL-9 production by ILC2s. We propose that epithelial alarmins IL-33 and TL1A, and IL-9^high^ ILC2s, function together in a sequential alarm system that is immediately activated after allergen exposure for rapid initiation of allergic airway inflammation.

IL-33 and TSLP have been well characterized as two major epithelium-derived cytokines in human lungs, and their therapeutic inhibition improves clinical symptoms in asthmatic patients (Brusselle and Koppelman, 2022; Cayrol and Girard, 2014, 2022; Hammad and Lambrecht, 2021; Howell et al., 2023; Lambrecht et al., 2019; Menzies-Gow et al., 2021; Wechsler et al., 2021). The identification of additional epithelium-derived cytokines cooperating with IL-33 and/or TSLP is thus of considerable scientific and clinical interest. Similar to IL-33 and TSLP, TL1A is expressed in basal cells in both upper and lower airways, and thus ideally located for cooperation with these two epithelial cytokines for induction of allergic type 2 inflammation in human airways. Previously, TL1A has been viewed as an inducible cytokine produced by activated immune cells and endothelial cells (Meylan et al., 2011; Migone et al., 2002; Richard et al., 2015). Our identification of TL1A as an epithelial cytokine that functions as an alarmin, similar to IL-33, thus significantly extends our understanding of the roles and mode of action of TL1A and provides an unappreciated upstream target for therapeutic intervention. Targeting epithelial cytokines that initiate allergic responses and act upstream in lung inflammatory cascades offers the potential to have more wide-ranging effects on downstream cytokines and immune cells (Brusselle and Koppelman, 2022; Howell et al., 2023). Antibodies targeting alarmins are therefore actively explored by drug companies because these biologics might improve asthma outcomes in a broader patient populations compared with antibodies against specific type 2 cytokines (Brusselle and Koppelman, 2022; Howell et al., 2023). Interestingly, anti-TL1A antibodies are currently under clinical development for inflammatory bowel diseases (Danese et al., 2021). In the future, the availability of a portfolio of monoclonal antibodies against different epithelial alarmins, including TL1A, could be useful for precision medicine, i.e., targeting the specific mechanisms driving an individual person’s asthmatic disease (Brusselle and Koppelman, 2022; Howell et al., 2023).

A remarkable property of the IL-33-TL1A-IL-9^high^ ILC2s alarm system is its rapid induction. We show that IL-33 and TL1A are released in BAL fluids shortly (15 min) after a single exposure of naïve WT mice to *A. alternata* and that IL-9 mRNA and IL-9^high^ ILC2s are induced in the lungs a few hours (3–6 h) after IL-33 and TL1A release. The rapid induction of IL-9 expression in naïve ILC2s is likely explained by the fact that in naïve mice, the chromatin in proximity to ILC2 effector genes, including the *Il9* locus, is developmentally programmed (or poised) for high-level transcription upon activation (Shih et al., 2016). We observed that IRF4, a critical transcription factor for induction of *Il9* mRNA in ILC2s (Mohapatra et al., 2016), was rapidly upregulated in an IL-33-dependent manner after allergen exposure. Moreover, activation of transcription factor STAT5, which is important for the induction of IL-9^high^ ILC2s by IL-33 and TL1A ex vivo, is known to occur rapidly upon cell activation. Thanks to the accessibility of the *Il9* promoter in naive ILC2s, induction of *Il9* mRNA by IRF4, pSTAT5, and other transcription factors presumably starts very quickly after allergen exposure.

A unique feature of the IL-33-TL1A-IL-9^high^ ILC2s alarm system is its transient nature. The IL-33-induced signal is transient because IL-33 is an exceptionally potent and tightly regulated alarmin (Cayrol and Girard, 2018, 2022). The protein is rapidly inactivated by oxidation or protein degradation, a few hours after its extracellular release (Cayrol et al., 2018; Cayrol and Girard, 2018, 2022; Cohen et al., 2015; Scott et al., 2018). Endogenous TL1A protein is also transiently released and the highest levels are found in BAL fluids during the first hour after allergen exposure and slowly decline thereafter. It remains to be determined how and in what form membrane-bound TL1A is released in vivo (full-length protein or shorter forms, associated or not with membrane fragments). Similar to IL-33 and TL1A release, the ILC9 phenotype is transient. IL-9 expression by lung ILC2s after IL-33 plus TL1A stimulation exhibits an “explosive” profile. Massive IL-9 production is induced shortly after activation (6–14 h) and rapidly terminated (24–48 h). We also observed a transient induction of IL-9 production after repeated treatments with *A. alternata*, suggesting that the “ILC9 burst” can occur again after repeated allergen exposure. The transient induction of the IL-9^high^ ILC9 phenotype by IL-33 and TL1A provides additional evidence for the high degree of phenotypic plasticity of ILC2s (Spits and Mjosberg, 2022).

Another important characteristic of the IL-33-TL1A-IL-9^high^ ILC2s alarm system is its exquisite sensitivity. We show that it is rapidly activated after a single exposure of naïve mice to a low dose of the fungal allergen *A. alternata*. The system is also highly potent. Tight regulation is thus needed to prevent lung pathology. As mentioned, the transient nature of the IL-33 and TL1A signals is likely to be a major mechanism of regulation. Transient induction of CD200 in ILC9 cells could also play a role. Lung ILC2s constitutively express CD200R, and CD200/CD200R signaling inhibits GATA3 expression in ILC2s and limits their activation (Shafiei-Jahani et al., 2021). The transient upregulation of CD200 in IL-9^high^ ILC2s could thus provide a negative feedback mechanism and contribute via autocrine signaling through CD200R to the transient downregulation of GATA3 and its target genes *Il9r* and *Il1rl1* in ILC9 cells.

We found that IL-9^high^ ILC2s maintained production of large amounts of type 2 cytokines IL-5 and IL-13, despite GATA3 downregulation, indicating that high IL-9 expression distinguishes a distinct multicytokine-producing state of activated ILC2s. Increased frequencies of lung eosinophils, 1 wk after a single i.n. exposure of naïve WT mice to IL-33 plus TL1A suggested an increased inflammatory potential of IL-9^high^ ILC2s. In agreement with this possibility, our adoptive transfer experiments showed that ILC9 cells had robust persistence in vivo and an increased capacity to initiate IL-5-dependent airway eosinophilia. Lung ILC2s express IL-9R, and IL-9-mediated autocrine signaling promotes activation and survival of ILC2s in vivo (Licona-Limon et al., 2013; Mohapatra et al., 2016; Turner et al., 2013; Wilhelm et al., 2011). We thus believe that an important role of the ILC9 burst and the IL-9 autocrine loop is to enhance the function and persistence of activated ILC2s after a tissue insult to increase the sensitivity and efficacy of the alarm system. In addition, the early production of IL-9 by ILC2s could also influence the function and/or survival of other lung immune cell types expressing the IL-9R (mast cells, B cells…) (Pajulas et al., 2023; Wilhelm et al., 2012). The enhanced pathogenicity of ILC9 cells in vivo suggests that responses mediated by these cells are likely to be detrimental during allergic airway inflammation and chronic airway diseases such as asthma. However, ILC9 cells could be beneficial in other contexts, particularly for tissue repair after viral or parasitic infection. We detected higher expression of amphiregulin (AREG) in ILC9 cells than in classical IL-33-activated ILC2s. ILC2-derived AREG is crucial for tissue-protective ILC2 responses after influenza or nematode infection (Monticelli et al., 2011; Tsou et al., 2022; Turner et al., 2013). The higher expression levels of AREG and the increased persistence of ILC9 cells in vivo could thus play important roles in lung tissue repair after infection or allergen exposure (Licona-Limon et al., 2013; Mohapatra et al., 2016; Monticelli et al., 2011; Turner et al., 2013).

Resident memory CD4^+^ T cells coproducing high levels of IL-9, IL-5, and IL-13, designated “Th9rm” cells, play a crucial role in allergic airway inflammation and airway hyperactivity after chronic allergen exposure in mice (Ulrich et al., 2022). Furthermore, populations of polyfunctional allergen-reactive ST2^+^ Th2 cells producing high levels of IL-9, IL-5, and IL-13, are specifically enriched in allergic individuals who develop asthma and display several features linked to pathogenicity and persistence, which may contribute to asthma pathogenesis (Seumois et al., 2020). The multicytokine-producing ILC9 phenotype of ILC2s is thus reminiscent of the phenotype of IL-9-expressing pathogenic Th2 cells in humans and mice (Micosse et al., 2019; Seumois et al., 2020; Ulrich et al., 2022). BATF, JunB, and IRF4 regulate *Il9* transcription in T cells (Fu et al., 2019; Jabeen et al., 2013; Staudt et al., 2010). Coordinated upregulation of BATF, JunB, and IRF4 in the IL-33-activated ILC2 proteome could thus explain our observation that treatment with IL-33 alone was sufficient for induction of IL-9 expression in ILC2s, as reported in previous studies (Mohapatra et al., 2016; Wilhelm et al., 2011). However, the full acquisition of the ILC9 phenotype with up to 98% of ILC2s producing high amounts of IL-9 required co-stimulation with TL1A. STAT5 and pSTAT5 levels were specifically increased after costimulation with IL-33/TL1A, and pSTAT5 was important for IL-9 expression in ILC9 cells. IL-2 was also required for induction of IL-9^high^ ILC2s ex vivo and in vivo. IL-2 is a potent inducer of STAT5 phosphorylation. Thus, cooperation between IL-33 and TL1A that increases intracellular levels of STAT5, and IL-2 that promotes STAT5 phosphorylation are likely to explain the high levels of pSTAT5 in ILC9 cells. In T cells, pSTAT5 acts as a pioneer transcription factor that promotes recruitment of BATF to the *Il9* locus (Fu et al., 2020). NFkB2, another regulator of IL-9 expression in T cells (Xiao et al., 2012), was also upregulated in ILC9 cells compared with IL-33-activated ILC2s (Fig. 3 B and Fig. S2 D). Increased recruitment of BATF/JunB/IRF4 complexes to the *Il9* locus facilitated by pSTAT5 and synergy with NF-kB2, after co-stimulation with IL-33 and TL1A, could contribute to the very high levels of IL-9 mRNA in ILC9 cells. Future studies based on chromatin immunoprecipitation assays will be required to confirm this possibility.

In conclusion, this study shows that TL1A is an epithelial alarmin rapidly released by airway epithelial cells after allergen exposure that cooperates with IL-33 during the onset of allergic airway inflammation. As an epithelial alarmin, TL1A acts upstream in lung inflammatory cascades and represents a bona fide target for therapeutic intervention. Together, our data provide additional evidence for the central role of airway epithelial cells in the initiation of allergic type 2 inflammation. Moreover, the identification of IL-33 and TL1A as two critical inducers of IL-9-producing ILC2s paves the way for a better understanding of the plasticity and roles of IL-9^high^ ILC2s in type 2 immunity and inflammatory diseases.

## Materials and methods

### Mice

C57BL/6 J (wild type) mice were purchased from Charles River Laboratories. *Rag2*^*−/−*^ mice on a C57BL/6 J background (B6.129-Rag2tm1Fwa) were obtained from EMMA (European Mouse Mutant Archive). *Il33*^*−/−*^ mice on a C57BL/6 J background (B6-Il-33GtIST10946B6–Tigm–Girard) have been previously described (Pichery et al., 2012). *IL-9-eGFP* fluorescent reporter mice (INFER IL-9 fluorescent reporter mice) were provided by R.A. Flavell (Yale University, New Haven, CT, USA) (Licona-Limon et al., 2013). *Red5* mice were a gift from R.M. Locksley (University of California, San Francisco, San Francisco, CA, USA). *Red5* mice are IL-5 reporter mice containing a tandem dimer red fluorescent protein (tdTomato) linked to a *Cre* element replacing the translation initiation site of the endogenous *Il5* gene (Nussbaum et al., 2013). Mice 6–12 wk of age were used for all experiments unless indicated otherwise; all animals were age- and sex-matched and then randomized into different groups. The exact number of animals used in individual experiments is indicated in figure legends. No statistical methods were used to predetermine sample size. The investigators were not blinded to allocation during experiments or outcome assessment. All mice were maintained in specific-pathogen-free animal facility at IPBS and were handled according to institutional guidelines under protocols approved by the French Ministry of Research and the FRBT (C2EA-01) animal care committee (projects APAFIS#00663.02, APAFIS#3873-2016020116301837v3, APAFIS#12812-2018031218075551v2, and APAFIS #37514-2022053019171556 v6).

### Administration of the fungal allergen *A. alternata*, cytokines, and antibodies

Mice were anesthetized by isoflurane inhalation followed by a single i.n. administration of 50 µl of *A. alternata* extract (12.5 µg; Greer Laboratories) or recombinant IL-33 (rIL-33, 1 µg; house-made [Cayrol et al., 2018]), and/or recombinant TL1A (rTL1A, 5 µg; R&D Systems) in PBS. Lungs and BAL fluids were collected at different time points (15 min, 1 h, 3 h, 6 h, 14 h, 24 h, 48 h) after the single i.n. treatment with *A. alternata* or cytokines for flow cytometry, Western blot, qPCR, and ELISA analyses. In some experiments, mice were treated by a single i.n. administration of *A. alternata* extract (12.5 µg), coadministrated with two doses (10 µg i.n. at t-12 h and 10 µg i.n. at t0) of the function-blocking anti-TL1A mAb L4G6 (Fang et al., 2008) (L4G6, # EMI006; Kerafast) or its isotype control (Armenian hamster IgG, clone PIP, #BE0260, RRID: AB_2687739; BioXCell) (Fig. 8, C–H), or the function-blocking anti-IL-2 mAb JES6-1A12 (# 503706, RRID: AB_11150775; Biolegend) or its isotype control (rat IgG2a, clone 2A3, # BE0089, RRID: AB_1107769; BioXCell) (Fig. S5 G). For in vivo imaging of endogenous IL-9^high^ ILC2s (Fig. 5, K and L), 100 µl of rIL-33 (1 µg) in PBS was injected i.p. for 6 consecutive days to expand lung ILC2s (Fig. S5 F), before a single i.n. administration of rIL-33 (1 µg) and/or rTL1A (5 µg) in PBS.

### Analysis of lung and BAL fluid samples

Upon exposure of the trachea, bronchoalveolar lavage fluid was obtained by slow injection of 1 ml PBS into the lung, which was subsequently recovered again in the syringe. The lavage was repeated twice. The fluid was centrifuged at 400 *g* at 4°C for 5 min. The supernatant was stored at −80°C for further measurement of cytokines, and cells were collected, stained, and analyzed by flow cytometry. Lung cell suspensions were obtained by lung digestion with 2 mg/ml collagenase D and 0.1 mg/ml DNAse I for 60 min at 37°C and mashed through a 70-μm cell strainer. In some experiments (Fig. 2 C), for better preservation of epithelial cell viability, lung digestion was performed with Dispase II (15 U/ml, 3 ml/lung, #17105041; Gibco) in the presence of low melting point agarose 2% for 45 min at RT, followed by incubation with 0.05 mg/ml DNAse I for 10 min at RT. Red blood cells were lysed after digestion by applying ACK (ammonium-chloride-potassium) lysis buffer for 1 or 2 min on BAL fluids or lung cells, respectively. Total cells in the lungs and BAL fluids were counted with a hemocytometer and stained for flow cytometry analysis.

### Analysis of IL-33 forms in BAL fluids

C57BL/6J wild type and *Il33*^*−/−*^ mice were anesthetized by isoflurane inhalation and treated i.n. with *A. alternata* (10 or 12.5 µg) in 50-μl PBS. BAL fluids were collected at selected time points after the challenge (5 or 15 min) by lavage with PBS (0.5 ml + 1 ml) containing protease inhibitor cocktail (Roche), 2 mM AEBSF, and 20 μM E64. For pull-down assay, protein G sepharose beads (60 μl; GE Healthcare) were coated with 1 μg of soluble IL-33 receptor (murine ST2-Fc chimera protein, produced in-house) overnight at 4°C in 1 ml PBS, washed with PBS containing protease inhibitor cocktail (Roche), 2 mM AEBSF, and 20 μM E64, and then blocked with 1% BSA for 1 h. BAL fluids were incubated with ST2-Fc-protein G sepharose beads for 3 h at 4°C or 2 h at room temperature in the presence of 10% fetal calf serum. After washing, the precipitates were eluted in 10 μl Laemmli buffer and loaded on 12% SDS-PAGE to identify endogenous IL-33 forms by immunoblot with goat antiserum to mouse IL-33-Cter (# AF3626, RRID: AB_884269; 1/1,000; R&D Systems). HRP-conjugated donkey anti-goat polyclonal antibodies (#V8051, RRID: AB_430838; 1/10,000; Promega) were used for detection. Recombinant murine IL-33_FL_, IL-33_102–266_, and IL-33_109–266_ were used as controls in the pull-down/immunoblot analyses.

### Isolation and culture of lung ILC2s

ILC2s were isolated from pooled lungs of IL-33-treated *Rag2*^−/−^ C57BL/6J mice as described (Schmitt et al., 2018). In brief, Lin^^−^^ cells were enriched by two successive rounds of depletion of Lin^+^ cells using biotin-conjugated antibodies to CD5, CD11b, CD19, CD45R/B220, Ly-6G/C (Gr-1), Ter119, and 7-4 and Easysep D magnetic particles (Mouse Hematopoietic progenitor cell enrichment kit, Stem cell Technologies). Subsequently, CD45^+^Lin^−^ cells were selected by using anti-mouse CD45 microbeads (Miltenyi Biotech). ILC2 phenotype was analyzed after purification by assessing the expression of lineage markers, CD90.2, Sca-1, IL-33R (ST2), CD25, ICOS, and KLRG1 using antibodies identified below. Lung CD45^+^Lin^−^ ILC2s were cultured for 4–6 days in 6-well plates at a density of 3 × 10^5^ cells/ml in RPMI medium supplemented with 10% fetal calf serum, 1% penicillin-streptomycin, 50 µM β-mercaptoethanol, and 20 ng/ml recombinant IL-2 (rIL-2). Cells were stimulated overnight (14 h) with different combinations of cytokines, used at the following concentrations: 20 ng/ml rIL-33_95–270_, 50 ng/ml rTL1A_72–270_, 50 ng/ml rIL-7, 50 ng/ml rTSLP, 10 ng/ml rTGF-β, and 10 ng/ml rIL-4. All cytokines were obtained from R&D Systems except rIL-33_95–270_, which was house-made as previously described (Cayrol et al., 2018). Flow cytometry analysis was performed before stimulation to assess the phenotype and frequency of ILC2s (typically ∼90% at day 0, the day of ILC2s isolation from the lungs, and >97% after 3–6 days of ex vivo culture in the presence of IL-2) (Fig. S2) (Schmitt et al., 2018). In some experiments (Fig. 3, I–K), increasing doses of a STAT5 inhibitor (STAT5i; CAS 285986-31-4; # 573108; Calbiochem) were added to ILC2s stimulated with rIL-33, rTL1A, and rIL-2.

### Flow cytometry

Single-cell suspensions were stained for 30 min at 4°C with combinations of the following antibodies, diluted in FcR block (CD16/CD32 clone 2.4G2, # 553142, RRID: AB_394657; BD Biosciences): CD45 (clone 30F11, # 557235, RRID: AB_396609; BD Biosciences and # 17-0451-82, RRID: AB_469392; eBioscience), SiglecF (clone E50-2440, # 552126, RRID: AB_394341; BD Biosciences), CD4 (clone GK1.5, # 11-0041-85, RRID: AB_464892; eBioscience), CD19 (clone eBio1D3, # 561740, RRID: AB_396681; BD Biosciences), CD45R (clone RA3-6B2, # 553088, RRID: AB_394618; BD biosciences), NK1.1 (clone PK136, # 11-5941-85, RRID: AB_465318; eBioscience and # 108739, RRID:AB_2562273; Biolegend), CD3 (clone 17A2, # 11-0032-82, RRID: AB_2572431; eBioscience and eF450 # 48-0032-82, RRID: AB_1272193; eBioscience), CD11b (clone M1/70, # 11-0112-85, RRID: AB_464936; eBioscience), Ter119 (clone Ter119, # 11-5921-85, RRID: AB_465311; eBioscience), Ly-6G/Ly6C (Gr-1, clone RB6-8C5, # 11-5931-81, RRID: AB_465313; eBioscience), FcεRIa (clone MAR-1, # 11-5898-85, RRID: AB_465308; eBioscience), CD11c (clone N418, # 11-0114-85, RRID: AB_464941; eBioscience and # 17-0114-82, RRID: AB_469346), CD90.2 (clone 53–2.1, # 561641, RRID: AB_10898013; BD Biosciences), Sca-1 (clone D7, # 17-5981-83, RRID: AB_469488; eBioscience), CD25 (clone PC61.5, # 48-0251-82, RRID: AB_10671550; eBioscience), CD127 (clone A7R34, # 12-1,271-82, RRID: AB_465844; eBioscience), KLRG1 (clone 2F1, # 17-5893-81, RRID: AB_469469; eBioscience), ICOS (clone 7E-17G9, # 12-9942-82, RRID: AB_466274; eBioscience), DR3 (clone 4C12, # 144405, RRID: AB_2561688; Biolegend), CD200 (clone OX-90, # 123807, RRID: AB_2275651; Biolegend), IL-33R (T1/ST2, clone DJ8, # 101001B, RRID: AB_947551; MD Biosciences), TL1A (clone Tandys1a, # 46-7911-82, RRID: AB_11217878; eBioscience), EpCAM (CD326, clone G8.8, # 118205, RRID: AB_1134176; BioLegend), CD31 (clone MEC13.3, # 102513; RRID: AB_493413; BioLegend), TCR beta (clone H57-597, # 48-5961-80, RRID:AB_11062012; eBioscience), and TCR gamma/delta (clone eBioGL3, # 48-5711-82, RRID:AB_2574071; eBioscience). Antibodies were diluted in FcR block (CD16/CD32 clone 2.4G2, # 553142, RRID: AB_394657; BD Biosciences) for 30 min at 4°C. For intracellular cytokine analysis, cells were restimulated for 4 h at 37°C with 50 ng/ml PMA and 500 ng/ml ionomycin in the presence of GolgiPlug, a protein transport inhibitor containing brefeldin A (1/1,000; BD Biosciences). In some experiments (Fig. 4, E, I, and J; and Fig. S3, A and D), cytokine analysis was performed after incubation with GolgiPlug (4 h at 37°C) without PMA/iono restimulation. Cells were fixed and permeabilized with the Foxp3 transcription factor buffer set (eBioscience) or 4% paraformaldehyde (PFA) for 20 min at 4°C followed by 0.5% Triton X-100 for 10 min at 4°C. Cells were then incubated with antibodies against IL-5 (clone TRFK5, # 12-7052-82, RRID: AB_763587; eBioscience), IL-13 (clone eBio13A, # 25-7133-80, RRID: AB_2573530; eBioscience), IL-9 (clone RM9A4, # 50-8091-82, RRID: AB_11218680; eBioscience), IRF4 (clone IRF4.3E4, # 12-9858-80, RRID: AB_10852721; eBioscience), and/or GATA3 (clone TWAJ, # 12-9966-42, RRID: AB_1963600; eBioscience) for 30 min at 4°C. Isotype-matched control antibodies and single-stain control samples were included. Dead cells were excluded by using viability dye eFluor506 (# 65-0866-14, 1/1,000; eBioscience). Samples were acquired on a LSRII or LSRFortessa flow cytometer using DiVa software (BD Biosciences) and analyzed using FlowJo software (Tree Star).

Cultured lung ILC2s were analyzed with the following markers: CD90.2, Sca-1, IL-33R (ST2), CD25, ICOS, KLRG1, and DR3. Lung ILCs were identified among lung single-cell suspensions as live Lin^−^ (lineage marker-negative: CD4^−^CD19^−^CD45R^−^NK1.1^−^CD3^−^CD11b^− ^CD11c^−^Ter119^−^Ly6G^−^FcεRI^−^) CD45^+^CD90.2^+^ single cells, as previously described (Lefrançais et al., 2014), and lung ILC2s as live IL-5^+^IL-13^+^ ILCs (Fig. S5A). We verified the absence of contamination of the IL-5^+^IL-13^+^ ILC2s and IL-9^high^ ILC2s populations by TCR^+^ cells (T cells and NKT cells) using anti-TCRβ and anti-TCRγδ antibodies (Fig. S5 B), and we confirmed the expression of IL-5 and IL-13 in Lin^−^CD3/TCR^−^NK1.1^−^CD45^+^CD90.2^+^ lung ILCs (Fig. S5 C) using antibodies against CD3/TCR and NK1.1 with a different fluorescence from the Lin cocktail (CD4, CD19, CD45R, CD11b, CD11c, Ter119, Ly6G, FcεRI), as suggested by Sadeghalvad et al. (2023). Lung and BAL eosinophils were identified as live CD45^+^Gr-1^low^CD11c^−^SiglecF^+^ single cells, lung endothelial cells as live CD31^+^CD45^−^ single cells, and lung epithelial cells as live Epcam^+^CD31^−^CD45^−^ single cells.

### TL1A expression vector, transfection, and allergen treatment

A pCMV6-mTL1A (Tnfsf15, NM_177371) expression vector allowing ectopic expression of the mouse TL1A protein (252 aa) tagged at its C-terminus with the epitope Myc-DDK (Flag), under the control of a CMV promoter (mTL1A-Flag vector), was obtained from Origene Technologies (# MR203224). Plasmid pcDNA3.1 (CMV-promoter) was used as a control vector. The U2OS epithelial cell line (# HTB-96; ATCC), an adherent epithelial cell line with 20–60% transfection efficiency was used for ectopic expression of mTL1A. U2OS cells grown in DMEM supplemented with 10% FBS and 1% penicillin-streptomycin were transiently transfected with mTL1A-Flag vector or control vector by using a phosphate calcium precipitation method. 2 days after transfection, cells were analyzed by immunofluorescence microscopy to verify TL1A expression. Cells were then treated with allergens, *A. alternata* extracts (20 μg/ml, 15 min) or bee venom phospholipase A2 (PLA2, #P9279, 100 μg/ml, 1 h; Sigma-Aldrich), and the supernatants were collected for TL1A ELISA analyses.

### Immunofluorescence microscopy on cultured cells

Cells were fixed for 15 min at room temperature in PBS containing 4% methanol-free PFA. Cells were then directly blocked with 1% bovine serum albumin in PBS and incubated for 1 h at room temperature with mAbs to mouse TL1A (rat IgG1 mAb, clone 293327, 2 μg/ml, # MAB7441; RRID: AB_2206977; R&D Systems) or DDK (Flag) epitope (rabbit mAb, clone TA592569S, 1 μg/ml, # TA592569; Origene). Cells were then washed three times and incubated for 1 h with Cy2- (or Cy3) conjugated donkey anti-rat IgG or Cy3-conjugated donkey anti-rabbit IgG secondary antibodies (1/500, # 712-225-153, RRID: AB_2340674, # 712-165-153 RRID: AB_2340667, and # 711-165-152, RRID: AB_2307443; Jackson ImmunoResearch). After extensive washing in PBS and counterstaining with DAPI, samples were air-dried and mounted in Mowiol. Images were visualized on a Nikon Eclipse TE300 fluorescence microscope equipped with a 40×/0.75 objective and captured through a Nikon digital camera DS-Fi3 (Nikon) using NIS-Elements D software (Nikon).

### Immunohistofluorescence of mouse lung tissue

Lungs were harvested after PBS and PFA 4% perfusion of mice under anesthesia, inflated with PFA, and fixed for 30 h at 4°C. Lungs were embedded in paraffin (Excelsior ES; Thermo Fisher Scientific) and sectioned at 4 μm with a microtome (RM2245; Leica). Sections were heated at 60°C for 1 h. After rehydration, sections were heated in AR6 buffer (# AR600; Akoya) using a Bio SB Tintoretriever for 15 min (program low pressure; 106–110°C) and then slowly cooled down at room temperature (RT) 30 min and washed twice (10 min) in distilled water. Sections were incubated for 10 min with Opal blocking solution (# ARD1001EA; Akoya). Anti-TL1A antibody (rat IgG1, clone 293327, #MAB7441; RRID: AB_2206977; R&D Systems), isotype control rat IgG1 (clone eBRG1 #25-4301-82; RRID: AB_470197; eBioscience), or isotype control rat IgG1 (rat anti-mouse CD62P, clone RB40.34, # 553741, RRID: AB_2254315; BD Pharmingen) was diluted at 10 µg/ml in Opal blocking solution and sections were incubated overnight at 4°C. Then sections were washed in PBS (3 × 10 min), incubated with a peroxidase-coupled donkey anti-rat IgG (# 712-035-153, RRID: AB_2340639; Jackson Immunoresearch) diluted at 4 µg/ml in Opal blocking solution 1 h at RT. After PBS wash (3 × 5 min), substrate TSA (Tyramide Signal Amplification) Vivid Fluorophore 650 (# 323273; Bio-techne) was diluted 1/1,500 in amplification diluent (# FP1498; Akoya) and incubated 15 min at RT. Sections were washed in PBS for 3 min and incubated with Opal blocking solution 10 min at RT, followed by goat anti-RAGE antibody (#AF1145; RRID: AB_354628; 2 µg/ml; R&D Systems) or goat anti-mIL-33 (#AF3626, RRID: AB_884269; 1 µg/ml; R&D Systems) 2 h at RT. Sections were washed in PBS (3 × 5 min) and incubated with Alexa 488 coupled bovine anti-goat IgG (# 805-545-180, RRID: AB_2340883; 7 µg/ml; Jackson Immunoresearch) diluted in PBS 1 h at RT. Sections were washed in PBS (3 × 5 min) and nuclei were counterstained with 4′,6-diamidino-2-phenylindole (# D9542; Sigma-Aldrich) and mounted with Mowiol. Photos were captured at high magnification, objective X100/1.40 oil M27 PlanApochromat (Zeiss) using an Axio Imager M2 microscope (Zeiss) with a CMOS ORCA Flash 4.0 LT digital camera C1144O (Hamamatsu). Zen pro software (Zeiss) was used to process images.

The anti-TL1A rat IgG1 antibody (clone 293327, #MAB7441; R&D Systems) used for immunostaining of lung tissue sections is a function-blocking anti-TL1A mAb (Malhotra et al., 2018) that we validated on TL1A transfectants (Fig. 7 G). The staining pattern of TL1A-expressing cells with the anti-TL1A mAb on 4 μm tissue sections (Fig. 2, D and E) was similar to the staining observed on TL1A transfectants permeabilized with Triton X100. No staining was observed on lung tissue sections with the two distinct rat IgG1 mAbs used as isotype controls for the anti-TL1A mAb (Fig. S1, D and E).

### Measurements of cytokines and LDH levels

Cytokine levels in culture supernatants were determined using mouse IL-9 ELISA ready set go (eBioscience), IL-5 ELISA MAX Deluxe Sets (BioLegend), and IL-13 Duoset ELISA (R&D Systems), according to the manufacturer’s instructions. The concentration of IL-9 and IL-33 in BAL fluids was analyzed using mouse IL-9 ELISA ready set go (eBioscience) and mouse IL-33 quantikine ELISA (R&D Systems). TL1A levels in BAL fluids and culture supernatants were determined with a modified mouse duoset TL1A ELISA (R&D Systems) by using the function-blocking anti-TL1A mAb L4G6 (clone L4G6, #EMI006; Kerafast) as a capture antibody and the neutralizing anti-TL1A mAb 820446 (clone 820446, # MAB1896; R&D Systems) for detection. Measurement of LDH activity released from damaged cells was performed using a Cytotoxicity Detection Kit (LDH release; # 11644793001; Roche). LDH release was analyzed by measuring the absorbance of the samples (BAL fluids) or determining cytotoxicity (cell supernatants; percentage LDH release induced by *A. alternata* or bee venom PLA2 compared with the maximum releasable LDH activity in cells treated with Triton-X100), according to the manufacturer’s instructions.

### Real-time PCR

Total RNA was extracted from cultured ILC2s or lung tissue with the RNeasy Mini Kit (Qiagen) and reverse transcribed with the SuperScript IV VILO cDNA synthesis kit (Invitrogen) according to the provider’s instructions. Quantitative RT-PCR was performed on the 7500 Real Time PCR system (Applied Biosystems) with the Power SYBR Green PCR Master Mix (Applied Biosystems). Primer sequences are available on request. The samples were normalized to expression levels of the HPRT housekeeping gene. Data were expressed relatively to the control (IL-2-stimulated ILC2s) using the 2^−ΔΔCT^ method to evaluate the fold induction between different conditions. To have an idea of the abundance of mRNA, data were expressed relative to HPRT mRNA quantity using the 2^−ΔCT^ method.

### Analysis of lung single-cell RNA-seq atlases

Single-cell RNA-seq analysis of TL1A (*Tnfsf15/TNFSF15)* expression in mouse and human lungs was performed using datasets generated by the LungMAP Consortium and obtained from the LungMAP database (http://www.lungmap.net). UMAP plots were extracted from data obtained by Guo et al. (2023) and Zepp et al. (2121). ShinyCell (Ouyang et al., 2021) was used to visualize the results. The LungMAP consortium [U01HL122642] and the LungMAP Data Coordinating Center (1U01HL122638) are funded by the National Heart, Lung, and Blood Institute (NHLBI). For single-cell RNA-seq analyses of *TNFSF15*, *IL33*, and *TSLP* expression in human healthy and asthmatic lung epithelium, t-SNE plots were extracted from data obtained by the human lung single-cell atlas (Vieira Braga et al., 2019), and downloaded from https://asthma.cellgeni.sanger.ac.uk.

### Western blot analysis

Proteins were fractionated by SDS-PAGE, electroblotted, and detected with primary antibodies to phosphorylated STAT5 (Phospho-Stat5 Rabbit mAb clone C71E5, 1/2,000, # 9314; RRID: AB_2302702; Cell Signaling Technology), JunB (Rabbit mAb clone C37F9, 1/2,000, # 3753; RRID: AB_2130002; Cell Signaling Technology), α-tubulin (mAb clone B-5-1-2, 1/6,000, # T5168; RRID: AB_477579; Sigma-Aldrich), or β-actin (mAb clone AC-15, # A5441; RRID: AB_476744; Sigma-Aldrich), followed by HRP-conjugated secondary antibodies, and finally an enhanced chemiluminescence kit (Amersham ECL Prime, GE Healthcare). Odyssey XF Imaging System (LI-COR) and Empiria Studio2.3 software were used for imaging Western blots.

### Adoptive cell transfer experiments

Lung ILC2s isolated from *Rag2*^−/−^ or *Red5* mice were maintained for 4 days in an IL-2-containing medium and stimulated for 14 h with rIL-33 alone (classical IL-33-activated ILC2s) or combined with rTL1A (ILC9s). After being extensively washed in PBS, activated cells were suspended in PBS (100 µl) and intravenously (i.v.) injected into the tail vein of wild-type host mice. For analyses at day 7, 5 × 10^5^ classical IL-33-activated ILC2s or ILC9s were transferred in separate host mice. For early time points analyses (1–4 h after adoptive cell transfer), classical IL-33-activated ILC2s or ILC9s were stained with cell trackers (CFSE or CTO, 10 µM for 15 min at 37°C) and 6 × 10^5^ of each cell type was simultaneously transferred in the same host.

### Lung intravital microscopy

Lung intravital microscopy was used to visualize IL-33-activated ILC2s (CFSE^+^) or ILC9s (CTO^+^) adoptively transferred in WT mice (2–3 mo of age) and endogenous IL-9-eGFP^+^ ILC2s in INFER IL-9 fluorescent reporter mice (6 mo of age). The previously published lung intravital microscopy method using an intercoastal thoracic window (Headley et al., 2016; Looney et al., 2011), was adapted at the IPBS CNRS-Université de Toulouse TRI platform. Vessel space was identified thanks to a retroorbital i.v. injection of Evans blue (100 µl of 1% wt/vol solution in PBS). Mice were anesthetized with ketamine and xylazine and secured to a microscope stage. A small tracheal cannula was inserted, sutured into place, and attached to a MiniVent mouse ventilator (Harvard Apparatus). Mice were ventilated with a tidal volume of 10 μl of compressed air (21% O_2_) per gram of mouse weight, a respiratory rate of 130–140 breaths per minute, and a positive-end expiratory pressure of 2–3 cm H_2_O. Isoflurane was continuously delivered to maintain anesthesia and 300 μl of 0.9% saline solution were i.p. administered in mice every hour for hydration. Mice were placed in the right lateral decubitus position and a small surgical incision was made to expose the rib cage. A second incision was then made into the intercostal space between ribs 4 and 5, through the parietal pleura, to expose the surface of the left lung lobe. A flanged thoracic window with an 8 mm coverslip was inserted between the ribs and secured to the stage using a set of optical posts and a 90° angle post clamp (Thor Labs). Suction (20–25 mmHg) was applied to gently immobilize the lung (Dexter Medical 0–250 mbar). Mice were placed in 30°C heated box during microscopy acquisition to maintain the body temperature and the 2-photon microscope objective was lowered over the thoracic window. Intravital imaging was performed using a Zeiss 7 MP upright multi-photon microscope equipped with a 20×/1.0 objective and a Ti-Sapphire femtosecond laser, Chameleon-Ultra II (Coherent Inc.) tuned to 820 nm. Evans Blue, second harmonic generation of collagen structure, CFSE, and CTO emission signals were detected thanks to the respective bandpass filters: FarRed (640–710), blue (SP485), green (500–550), and red (565–610). We captured a 9 mm^2^ x-y surface area at 900 μm z depth, capturing a complete image every 1 min for 120 min. Images were analyzed using Imaris 7.6.1 software (Bitplane).

### Multiphoton microscopy after lung tissue clearing

After injection of Evans Blue to label vessels, lungs of activated INFER IL-9 fluorescent reporter mice were fixed overnight with 4% PFA at 4°C. Dehydration, delipidation, and clarification of lungs were performed at 37°C under slight agitation before microscopy as previously described (Jing et al., 2018). Briefly, lungs were delipidated by making successive baths of tert-butanol + 3% Quadrol (30%, 50%, and 70% for 2, 4, and 4 h respectively). Following this step, a dehydration step was carried out by incubation in 70% tert-butanol-30% PEG for 24 h. Finally, for clarification, incubation in a 75% benzyl-benzoate–25% PEG solution for 12 h was performed. All products were purchased from Sigma-Aldrich. Cleared lungs were maintained between a slide and a cover-slide in a 2.5 mm thick imaging chamber (CoverWell; Thermo Fischer Scientific) filled with ethylcinnamate. For multiphoton microscopy, Evans Blue, second harmonic generation of collagen structure and eGFP emission signals were detected thanks to the respective bandpass filters: FarRed (640–710), blue (SP485), and green (500–550). We acquired TileScan of 9 mm^2^ x-y surface area at 900 μm z depth. Images were analyzed using Imaris 7.6.1 software (Bitplane).

### Sample preparation for mass spectrometry (MS)

Lung ILC2s were incubated with cytokines overnight at 37°C. Proteins were extracted by cell lysis in 2% SDS, 62.5 mM Tris, pH 6.8, followed by sonication using a Bioruptor. Protein concentrations were determined using a detergent-compatible assay (DC assay; Bio-Rad) and total protein amounts were adjusted across samples. Cysteine residues were reduced by addition of 25 mM final of dithiothreitol for 5 min at 95°C, and alkylated by addition of iodoacetamide at a final concentration of 90 mM for 30 min at room temperature in the dark. For three biological replicates, protein samples were migrated briefly on SDS-PAGE gel and a single slice containing the whole protein sample was excised, and processed for protein digestion. To increase proteomic depth, three additional biological replicates were further analyzed following fractionation into five gel bands through SDS-PAGE. Gel bands were washed consecutively with 100 mM ammonium bicarbonate and with 100 mM ammonium bicarbonate–acetonitrile (1:1). Proteins were in-gel-digested overnight at 37°C using 0.6 μg modified sequencing grade trypsin (Promega) in 50 mM ammonium bicarbonate. The resulting peptides were extracted from the gel by consecutive rounds of incubation (15 min, 37°C) in 50 mM ammonium bicarbonate and 10% formic acid–acetonitrile (1:1), dried in a speed-vac concentrator, and resuspended in 5% acetonitrile and 0.05% TFA for nanoLC-MS/MS analysis.

### NanoLC-MS/MS analysis

Peptides were analyzed by nanoscale liquid chromatography (nanoLC) coupled to tandem MS (MS/MS) using an UltiMate 3000 RSLCnano system (Dionex) coupled to an Orbitrap-Velos or Orbitrap Fusion mass spectrometer (Thermo Fisher Scientific). Peptides were separated on a C-18 column (75 μm inner diameter × 50 cm; packed in-house with Reprosil C18) equilibrated in 95% solvent A (5% acetonitrile and 0.2% formic acid) and 5% solvent B (80% acetonitrile and 0.2% formic acid) using a 5–50% gradient of solvent B in 365 min (unfractionated samples) or 160 min (gel bands fractions) at 300 nl/min flow rate. The mass spectrometer was operated in data-dependent acquisition mode with the XCalibur software. MS survey scans were acquired in the Orbitrap with a resolution of 120,000 (Orbitrap Fusion) or 60,000 (Orbitrap-Velos). Peptide ions were automatically selected and sequenced either by CID (collision-induced dissociation) in the linear ion trap or by HCD (higher-energy collisional dissociation) in the c-trap with analysis of the fragments in the Orbitrap with a resolution of 30,000 (Orbitrap Fusion) or in the linear ion trap (Orbitrap-Velos).

### Protein identification and quantification

Raw MS files were processed with MaxQuant software (version 1.5.2.8) for database search with the Andromeda search engine and quantitative analysis. Data were searched against *Mus musculus* entries in the UniProtKB/Swiss-Prot protein database and the set of common contaminants provided by MaxQuant. Carbamidomethylation of cysteines was set as a fixed modification, whereas oxidation of methionine and protein N-terminal acetylation were set as variable modifications. The specificity of trypsin digestion was set for cleavage after K or R, and two missed trypsin cleavage sites were allowed. The precursor mass tolerance was set to 20 ppm for the first search and 4.5 ppm for the main Andromeda database search. The mass tolerance in tandem MS mode was set to 20 mmu. The minimum peptide length was set to seven amino acids, and the minimum number of unique or razor peptides to one. Andromeda results were validated by the target decoy approach using a reverse database at both peptide and protein false-discovery rate of 1% at both the PSM and protein levels. For label-free relative quantification of the proteins across biological replicates and stimulation time points, the match between runs option of MaxQuant was enabled with a match time window of 1 min to allow cross-assignment of MS features detected in the different runs, after alignment of the runs with a time window of 20 min. Statistical analysis of the proteomic data was performed in the Perseus software. Protein abundance values (LFQ intensities) were log_2_-transformed, data were filtered (only proteins quantified with at least 50% of valid LFQ values in at least one biological group were retained), and the missing values were imputed for each analytical run with a noise value defined as the 1% lower percentile of the protein LFQ values distribution. Differentially abundant proteins were selected using a Student’s *t* test (P < 0.05) and a threshold on the fold change (FC) between the stimulated and non-stimulated conditions (abs[log_2_ FC] >1). Analysis of IL-33-activated ILC2s revealed that cytokines IL-5 and IL-13 were among the most induced proteins, highlighting the reliability of direct measurements of ILC2s proteins through large-scale proteomic analyses.

### Statistical analysis

All experiments comparing treatment groups were made using randomly assigned littermates without investigator blinding. Comparisons among mice of different litters were made using age- and gender-matched cohorts. Results from independent experiments performed similarly were pooled. All data points reflect biological replicates; technical replicates were averaged to yield a single value for analysis. No data were excluded. All data were analyzed using Prism7 (Graph Pad Software). A P value of <0.05 was considered significant. An unpaired two-tailed Student’s *t* test was used for the comparison of independent experimental groups. For experiments containing more than two groups, one-way ANOVA followed by Tukey’s test was performed. Statistical details for figures (including the number of samples or mice per group [*n*], details of experimental replicates, and statistical tests done) are described in the legends.

### Online supplemental material

Fig. S1 shows the single-cell RNA-seq analysis of IL33 and TSLP expression in human lungs, and the gating strategy used for the analysis of mouse lung epithelial cells by flow cytometry. Fig. S2 shows the phenotypic analysis of cultured mouse lung ILC2s by flow cytometry and the proteomic analyses of lung ILC2s stimulated ex vivo with IL-33 and/or TL1A. Fig. S3 demonstrates that IL-33 and TL1A synergistically induce IL-9-producing ILC2s ex vivo. Fig. S4 shows that IL-33 and TL1A induce phenotypic changes in cultured lung ILC2s at the protein and mRNA levels. Fig. S5 shows the gating strategy of live lung IL-9^high^ ILC2s and the synergistic induction of IL-9-producing ILC2s by IL-33 and TL1A in vivo. Video 1 shows that endogenous IL-9-producing ILC2s accumulate around blood vessels after IL33/TL1A treatment in vivo. Video 2 shows that endogenous IL-9-producing ILC2s migrate along collagen fibers after IL33/TL1A treatment in vivo. Video 3 shows that endogenous IL-9-producing ILC2s are not present in PBS-treated mice. Video 4 shows that adoptively transferred ILC2s and ILC9s are equally recruited to the lung and exhibit an ameboid-like mode of migration. Table S1 lists the proteins identified in the large-scale proteomic analyses of lung ILC2s stimulated ex vivo with IL-33 and/or TL1A.

## Supplementary Material

Table S1 lists the proteins identified in the large-scale proteomic analyses of lung ILC2s stimulated ex vivo with IL-33 and/or TL1A.

SourceData F3 is the source file for Fig. 3.

SourceData F6 is the source file for Fig. 6.

SourceData FS3 is the source file for Fig. S3.

## Data Availability

The MS proteomics data have been deposited to the ProteomeXchange Consortium with the dataset identifier PXD041987. The authors declare that all other data supporting the findings of this study are available within the paper and its supplementary information files.
