# Supplementary figures and images for: TL1A is an epithelial alarmin that cooperates with IL-33 for initiation of allergic airway inflammation
Source: J Exp Med. 2024 Apr 10;221(6):e20231236. doi: 10.1084/jem.20231236 (PMC11010340; doi:10.1084/jem.20231236)

Source Data Fig 3H

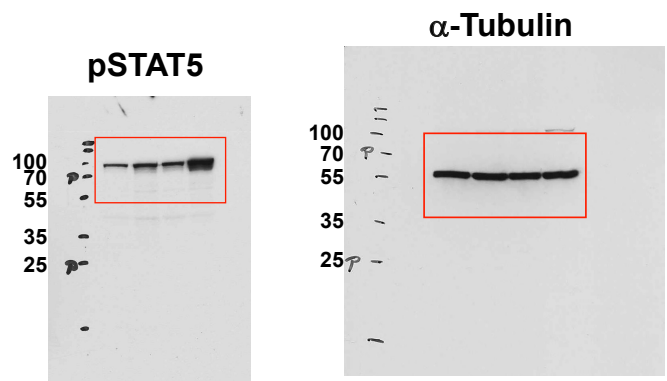

Source Data Fig 3I

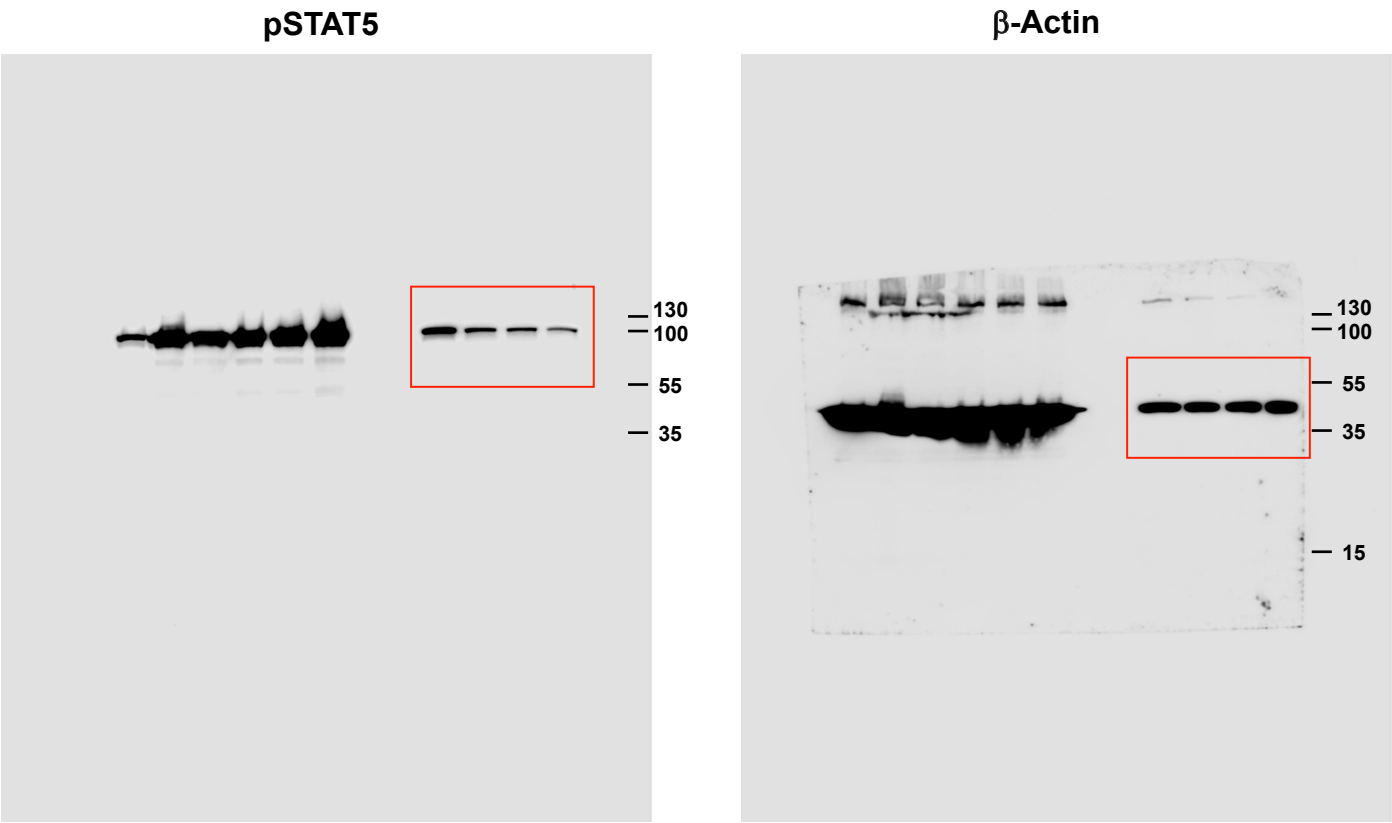

Supplement: SourceData F3 — is the source file for Fig. 3. [file JEM_20231236_SourceDataF3.pdf]

SourceData Fig 6C

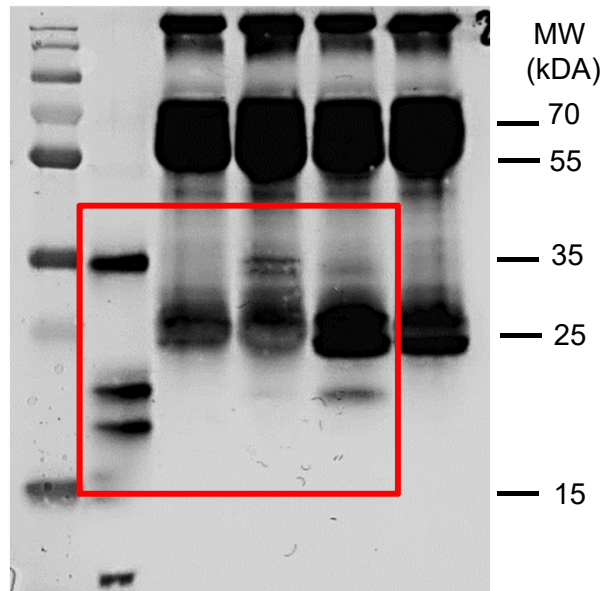

SourceData Fig 6D

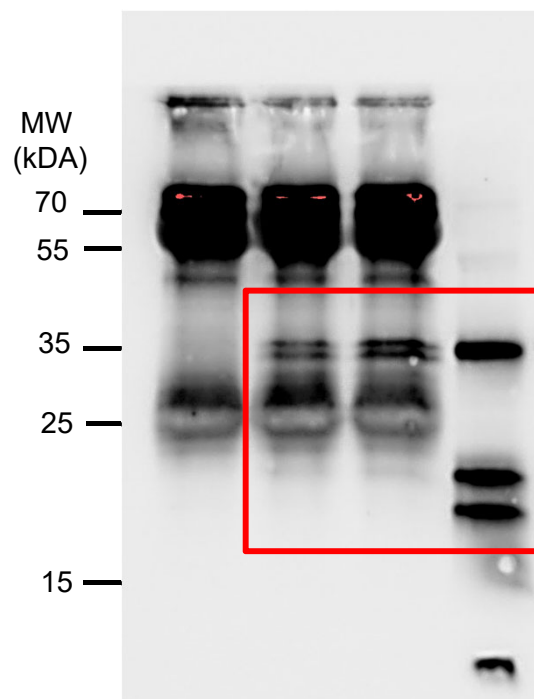

Supplement: SourceData F6 — is the source file for Fig. 6. [file JEM_20231236_SourceDataF6.pdf]

Source Data Fig S3E

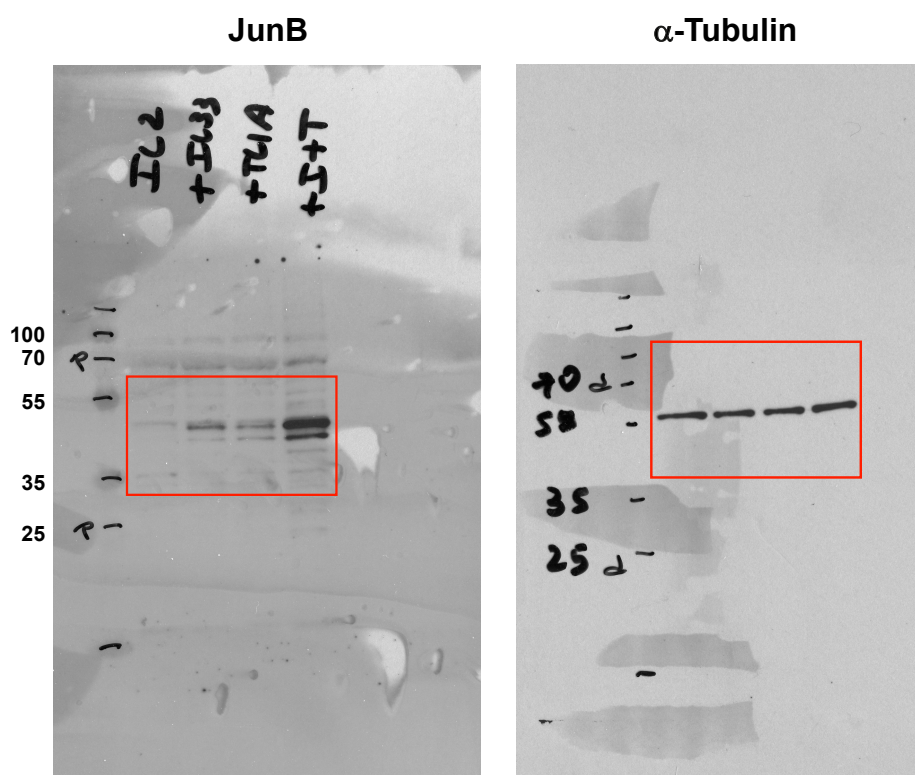

Supplement: SourceData FS3 — is the source file for Fig. S3. [file JEM_20231236_SourceDataFS3.pdf]
